# 2‐Amino‐4‐aryl‐5‐oxo‐4,5‐dihydropyrano[3,2‐*c*]chromene‐3‐carbonitriles with Microtubule‐Disruptive, Centrosome‐Declustering, and Antiangiogenic Effects *in vitro* and *in vivo*

**DOI:** 10.1002/cmdc.202200064

**Published:** 2022-03-16

**Authors:** Leonhard H. F. Köhler, Sebastian Reich, Gerrit Begemann, Rainer Schobert, Bernhard Biersack

**Affiliations:** ^1^ Organic Chemistry Laboratory University of Bayreuth Universitätsstraße 30 95447 Bayreuth Germany; ^2^ Department of Biology University of Bayreuth Universitätsstraße 30 95447 Bayreuth Germany

**Keywords:** Pyrano[3,2-*c*]chromene, Centrosome de-clustering, Antiangiogenic agents, Anticancer drugs

## Abstract

A series of fifteen 2‐amino‐4‐aryl‐5‐oxo‐4,5‐dihydropyrano[3,2‐*c*]chromene‐3‐carbonitriles (**1 a**–**o**) were synthesized via a three‐component reaction of 4‐hydroxycoumarin, malononitrile, and diversely substituted benzaldehydes or pyridine carbaldehydes. The compounds were tested for anticancer activities against a panel of eight human tumor cell lines. A few derivatives with high antiproliferative activities and different cancer cell specificity were identified and investigated for their modes of action. They led to microtubule disruption, centrosome de‐clustering and G2/M cell cycle arrest in 518 A2 melanoma cells. They also showed anti‐angiogenic effects *in vitro* and *in vivo*.

## Graphical abstract

**De‐clustering cancer**: A new 2‐amino‐4‐aryl‐5‐oxo‐4,5‐dihydropyrano[3,2‐*c*]chromene‐3‐carbonitrile with superior anticancer activity was identified. While selectively targeting cancer cells through centrosome de‐clustering, angiogenesis was significantly decreased *in vitro* and *in vivo*.

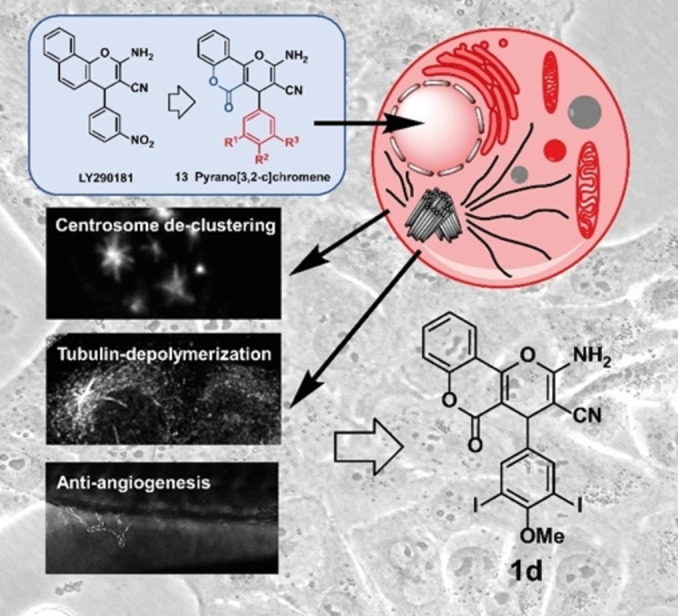

## Introduction

**Scheme 1:**
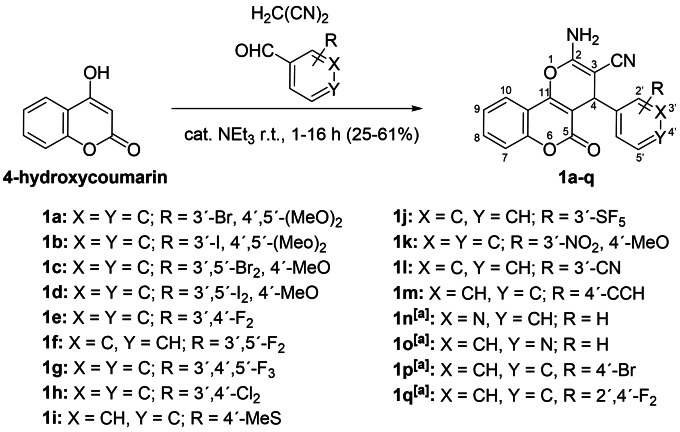
Syntheses of 2‐amino‐4‐aryl‐5‐oxo‐4,5‐dihydropyrano[3,2‐*c*]chromene‐3‐carbonitriles **1**.^[a]^ El‐Agrody et al.^[10]^

The development of new drugs is mainly based on the synthesis and screening of compound libraries, and on the elucidation of target‐ligand interactions, structure‐activity relationships (SAR) and target/disease selectivities.^[1]^ When following a target‐oriented strategy, one‐pot multi‐component reactions (MCR) are an excellent approach to the synthesis of large libraries of compounds with systematically varied substituents.^[2]^ Prominent examples of such multi‐component reactions often‐used for drug development are those named after Ugi, Biginelli and Van Leusen, and modifications thereof.^[3]^ The coumarin (1,2‐benzopyrone) scaffold is found in quite a few biologically active compounds including anticancer agents.^[4]^ 4‐Hydroxycoumarin, in particular, emerged as an important antitumoral pharmacophore (Scheme 1).^[5]^ It is fortunate that 4‐hydroxycoumarin reacts readily in one‐pot three‐component reactions with aryl aldehydes and malononitrile to give pyranochromene derivatives, which were frequently found active against cancer cells, especially when carrying halogen substituents.^[6]^ Analogous reactions using naphthol and phenol derivatives instead of 4‐hydroxycoumarin previously led to a plethora of similar anticancer active compounds.[^7^, ^8^, ^9^] In continuation of our recent work on anticancer active pyrans derived from 1‐naphthol and hydroxyquinoline, we now submitted the most promising substituted aryl aldehydes of these series (e. g., such with methoxyphenyl and fluorinated phenyl residues) to MCR with malononitrile and 4‐hydroxycoumarin. This afforded a series of new 2‐amino‐4‐aryl‐5‐oxo‐4,5‐dihydropyrano[3,2‐*c*]chromene‐3‐carbonitriles, whose anticancer activities and modes of action were assessed in detail.[^8^, ^9^] In particular, effects on microtubules, cellular morphology, cell cycle arrest, CDK, and angiogenesis were studied in this work.

## Results and Discussion

### Synthesis

The test compounds **1 a**–**q** were prepared from a mixture of 4‐hydroxycoumarin, malononitrile, the corresponding aryl aldehyde, and a catalytic amount of triethyl amine in acetonitrile (Scheme 1). The new compounds **1 a**–**m** were obtained as colorless solids in low to moderate yields. NMR, IR, and MS analyses confirmed the proposed structures.

### Antiproliferative activity

The compounds **1 a**–**o** were initially tested for their antiproliferative activity against a panel of eight tumor cell lines from five different entities and one endothelial hybrid cell line (EA.hy926) using MTT assays (Table 1). The previously published compounds **1 p** and **1 q** served as reference compounds but showed no activity in our experiments in the tested concentration range.^[10]^ Compounds **1 a**–**d** showed generally considerable antiproliferative activities with IC_50_ values in the low one‐digit micromolar range. The bromo‐derivatives **1 a** and **1 c** were slightly more active than the iodo analogs **1 b** and **1 d**. 3,5‐Dibromo‐4‐methoxyphenyl derivative **1 c** was especially active against HT‐29 colon carcinoma cells (IC_50_=0.5 μM).

**Table 1:** Inhibitory concentrations IC_50_^[a]^ [μm] of test compounds **1 a**–**q** when applied to 518 A2 melanoma, KB‐V1^Vbl^ MDR cervix carcinoma (treated with and without 1 μm verapamil), U‐87 MG likely glioblastoma, MCF‐7 breast carcinoma, HT‐29, HCT‐116 and HCT‐116p53−/− (p53 knockout mutant) colon carcinoma, EA.hy926 endothelial hybrid cells, and HDFa human dermal fibroblasts.

	EA.hy926	518 A2	HCT‐116	HCT‐116 p53−/−	U87	HT‐29	KB−V1^Vb.^	KB−V1^Vbl.^ [1 μm VER]	MCF‐7	HDFa
**1 a**	1.8±0.1	1.9±0.1	3.2±0.1	2.2±0.2	4.2±0.2	6.4±0.9	4.9±0.3	4.2±0.2	1.9±0.08	>100
**1 b**	6.2±0.2	2.9±0.1	6.7 ±0.2	1.7±0.16	12.3±1.4	5.6±0.3	7.8±0.8	7.0±1.4	5.8±0.2	–
**1 c**	1.0±0.06	1.8±0.2	1.5±0.05	1.9±0.1	3.4±0.2	0.5±0.05	3.0±0.2	2.8±0.2	1.1±0.1	>100
**1 d**	2.8±0.07	1.5±0.1	3.1±0.1	2.9±0.06	5.5±0.3	2.5±0.2	3.7±0.06	3.5±0.08	3.4±0.2	>100
**1 e**	>50	–	>50	>50	>50	>50	>50	–	–	–
**1 f**	8.8±0.3	6.7±0.5	7.8±0.6	9.8±0.4	12.6±2.2	13.7±1.5	21.2±2.8	7.4±1.4	5.8±0.2	–
**1 g**	>50	>50	>50	>50	>50	>50	29.3±2.0	>50	>50	–
**1 h**	7.0±0.5	>50	18.2±1.4	42±1.8	49.1±7.1	1.5±0.2	>50	>50	>50	–
**1 i**	–	–	>50	>50	–	>50	–	–	–	–
**1 j**	0.15±0.02	>50	>50	0.04±0.008	>50	0.4±0.02	>50	>50	>50	–
**1 k**	–	–	>50	>50	–	>50	–	–	–	–
**1 l**	10.2±0.7	13.3±1.0	15.3±1.0	16.6±3.3	15.7±1.7	35.1±3.6	14.6±1.2	2.9±0.3	14.4±0.9	–
**1 m**	>50	>50	13.5±1.2	27.4±1.2	7.4±0.8	>50	>50	>50	>50	–
**1 n**	–	–	–	>50	>50	–	>50	–	–	–
**1 o**	–	–	>50	>50	>50	>50	>50	–	–	–
**1 p**	–	>50	–	–	–	–	>50	>50	>50	–
**1 q**	–	>50	–	–	–	–	>50	>50	>50	–

[a] Values are the means of at least four independent experiments (±SD). They were derived from concentration‐response curves obtained by measuring the percentage of vital cells relative to untreated controls after 72 h using MTT‐assays.

Among the fluorophenyl derivatives, only 3,5‐difluorophenyl compound **1 f** exhibited moderate antiproliferative activities while the 3,4‐difluorophenyl and 3,4,5‐trifluorophenyl derivatives **1 e** and **1 g** were virtually inactive. In contrast to the inactive compound **1 e**, its dichlorophenyl analog **1 h** was active against HT‐29, EA.hy926 and HCT‐116 cells, while it remained inactive against 518 A2, KB‐V1 and MCF‐7 cells. Hence, **1 h** showed a certain degree of tumor type specificity. Particularly interesting activities and selectivities were observed for the 3‐pentafluorothiophenyl derivative **1 j**. It was highly active against EA.hy926, HCT‐116 p53−/−, and HT‐29 cells (IC_50_=0.15, 0.04, and 0.4 μM, respectively), while it was inactive against the other tumor cell lines. In contrast to that, the 3‐cyanophenyl derivative **1 l** showed moderate but unspecific activity against all tested cancer cell lines. Interestingly, the 4‐ethynylphenyl derivative **1 m**, which was designed for localization assays (see below), showed moderate activity only against HCT‐116, HCT‐116 p53−/−, and U87 cells. The 4‐methylthiophenyl analog **1 i**, the 3‐nitro‐4‐methoxyphenyl analog **1 k**, and the pyridyl derivatives **1 n** and **1 o** showed no antiproliferative activities below concentrations of 50 μM. Some of the compounds, e. g., **1 f**, **1 g** and **1 l**, might be a substrate of efflux transporter P‐gp, as cells treated with the P‐gp inhibitor verapamil (VER) prior to the application of these compounds gave rise to significantly lower IC_50_ values when compared to those measured in the absence of verapamil. By contrast, **1 a**–**d** displayed no significant difference in IC_50_ values of verapamil treated and untreated KB‐V1 cervix carcinoma cells. They are thus unlikely to be P‐gp substrates, which fact might contribute to their superior activity. To estimate their selectivity for cancer cells the three most active compounds **1 a**, **1 c** and **1 d** were also tested on non‐malignant adult human dermal fibroblasts (HDFa) via MTT assays. The selectivity index (SI) for all tested cancer cell lines was calculated from the ratio of the average IC_50_ value and that of HDFa cells (SI=IC_50_ HDFa/average IC_50_ of all cancer cell lines). Since all three compounds showed high SI values (29.3 for **1 a**, 52.9 for **1 c**, 31.1 for **1 d**), they can be considered as selective for cancer cells (Table S1, Supporting Information).^[11]^

The three most active compounds **1 a** (3,5‐dibromo‐4‐methoxy motif), **1 c** (3‐bromo‐4,5‐dimethoxy motif) and **1 d** (3,5‐diiodo‐4‐methoxy motif) were selected for further mechanistic studies. Despite its selectivity for and low IC_50_ values against EA.hy926, HCT‐116p53−/− and HT‐29 cells, compound **1 j** was excluded because of its lower average cytotoxicity and poor efficacy against the mainly used 518 A2 melanoma cell line.

### Effect on tubulin polymerisation and the microtubules

**Figure 1:**
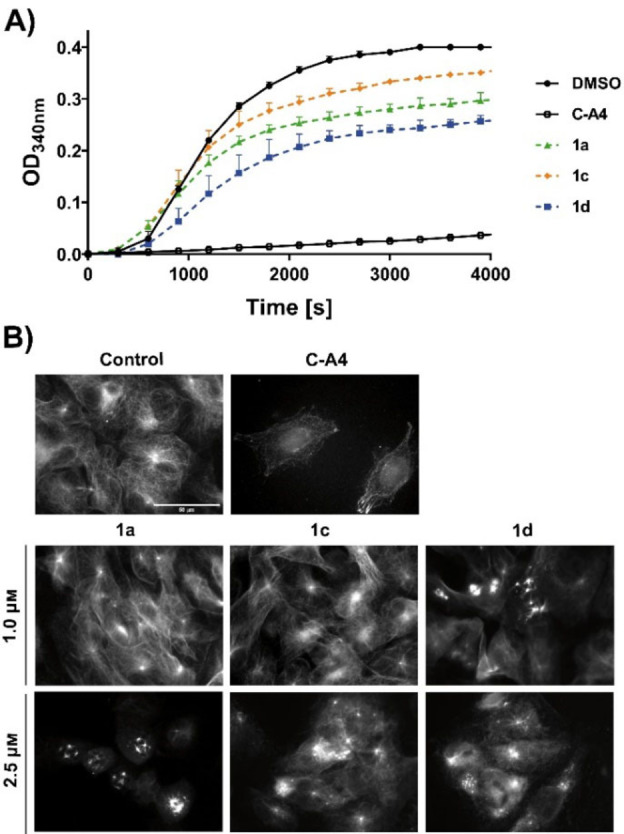
(A) Effect of compounds **1 a**, **1 c** and **1 d** (5 μm) on *in vitro* tubulin polymerisation as determined by a turbidimetric assay. Vehicle (DMSO) and C−A4 (5 μm) served as controls. Data are representative of two independent experiments and quoted as means ±SD. (B) Images illustrating tubulin cytoskeleton stained for α‐tubulin in 518 A2 melanoma cells after 24 h incubation with substances **1 a**, **1 c** and **1 d** (1 and 2.5 μm). Negative controls were treated with an equivalent amount of vehicle (DMSO) and positive controls were treated with 100 nm C−A4. Images are representative of at least three independent experiments. Magnification 630×.

The superior cytotoxicity of the methoxyphenyl derivatives **1 a**, **1 c** and **1 d**, which remotely structurally resemble combretastatin A‐4 (C−A4), may be due to a C−A4‐like interaction with cellular tubulin.^[12]^ Therefore, their effect on the *in vitro* polymerisation of tubulin was investigated (Figure 1A). As a component of the cytoskeleton, microtubules are essential for a variety of cellular processes including cell division, motility, transport, and structure stability, making them a promising target for anticancer therapeutics.^[13]^ Interestingly, compound **1 d** caused the strongest inhibition of the polymerisation of purified tubulin, even though its cytotoxicity was lower than that of **1 c**, suggesting that cellular targets other than tubulin might also play a role in cytotoxicity. To investigate the effects on tubulin within living cancer cells, the microtubules of 518 A2 melanoma cells were fluorescently stained 24 h after treatment with 1 and 2.5 μM of **1 a**, **1 c** or **1 d** (Figure 1B). Vehicle treated cells had a well‐organized and structured tubulin‐cytoskeleton, contrary to C−A4 (0.1 μM) treated cells, which had no intact microtubules except for some fragments. At concentrations of 1 μM, only **1 d** showed a slight destabilizing effect, apparent from a reduction and shortening of the microtubules. At a concentration of 2.5 μM, **1 a** and **1 c** also led to a strong decrease and fragmentation of intact microtubules, confirming the results of the polymerisation inhibition tests *in vitro*.

### Centrosomal de‐clustering

**Figure 2:**
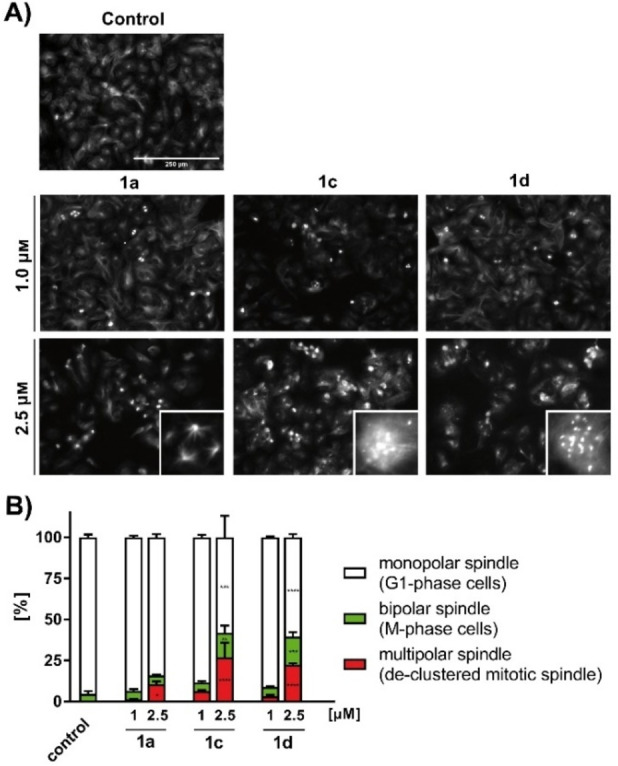
(A) Effect of compounds **1 a**, **1 c** and **1 d** (1 and 2.5 μm) on spindle apparatus formation and accumulation of multipolar spindles with α‐tubulin staining. Vehicle (DMSO) served as a control. Images are representative of at least three independent experiments. Magnification 100×. The insets show enlarged image sections with multipolar spindles (B) Histograms represent the percentage of cells with bipolar spindle apparatus (green) displaying cells in mitotic phase and multipolar spindle apparatus (red) displaying defective declustered spindles. 500–800 cells were counted per condition of 3 independent experiments. The significance was given as *p<0.05; **p<0.01; ***p<0.001; ****p<0.0001 against control for each concentration, One‐way ANOVA with Dunnett's multiple comparison test (GraphPad Prism 7).

In addition to the effects on the cytoskeleton, we observed an increase in bipolar and multipolar mitotic spindles for all three compounds (Figure 2A). After treatment with 2.5 μM of compound, the number of cells with bipolar spindles were slightly increased for **1 c** (10 %) and **1 d** (13 %), whereas cells with multipolar spindles increased by 11 % for **1 a**, by 22 % for **1 c** and by 26 % for **1 d** (Figure 2B). The increase in bipolar mitotic spindles indicates that cells are prevalent in prometa‐ or metaphase, which may be the consequence of an improper chromosome alignment resulting in an M phase spindle checkpoint arrest.^[14]^ Unlike healthy somatic cells, most tumor cells have multiple centrosomes, which would normally lead to the formation of multiple mitotic spindles, resulting in deficient chromosome segregation and cell death.^[15]^ Therefore, inhibition of centrosome clustering with the resulting induction of multipolar spindles and subsequent cell death would selectively affect tumor cells.^[16]^ As already described for other antimitotic drugs, compounds **1 a**, **1 c** and **1 d** induced a significant increase in multipolar mitotic spindles, suggesting a mode of action through interference with spindle tension and subsequent inhibition of centrosome clustering.^[17]^ Furthermore, it is possible that **1 a**, **1 c** and **1 d** inhibit other key enzymes involved in centrosome‐cluster formation.^[18]^

In recent studies the inhibition of centrosome clustering has been described as a promising approach for selectively targeting cancer cells.^[19]^ As cancer cells rely on centrosome clustering for survival, whereas healthy cells with normal centrosome complement are hardly affected, centrosome declustering could offer an opportunity for chemotherapy with few side effects.

### Effects on cell cycle and CDK1/CyclinA2 activity

**Figure 3:**
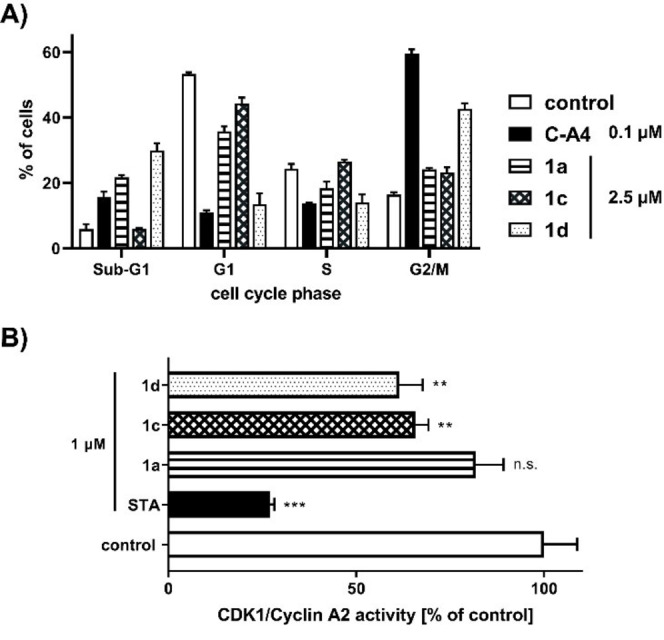
(A) Ratio of cells in different cycle phases of 518 A2 melanoma cells after 12 h incubation with compounds **1 a**, **1 c** and **1 d** (2.5 μm). Negative controls were treated with an equivalent amount of solvent (DMSO) and positive controls with C−A4 (100 nm). The assay was performed in triplicate and quoted as means ±SD. (B) Inhibition of the cell cycle regulating CDK1/Cyclin A2 complex assessed by Kinase Enzyme Assay Kit (Promega). **1 a**, **1 c**, **1 d** and positive control staurosporine (STA; 1 μm) were tested in duplicate and DMSO served as negative control. The significance was given as *p<0.05; **p<0.01; ***p<0.001; ****p<0.0001 against control, Two‐way ANOVA (A), One‐way ANOVA (B), with Dunnett's multiple comparison test (GraphPad Prism 7).

To investigate this more closely, substance‐treated cells were analysed by FACS and the percentage of cells in the respective cell cycle phase was determined. Upon treatment of 518 A2 melanoma cells with 2.5 μM of **1 a**, **1 c** or **1 d** for 12 h, an arrest in the G2/M‐phase was observed for all three substances, most pronounced so for **1 d** with an increase of cells in this phase of 26 % (Figure 3A). This supports the assumption that the cell cycle is also influenced by the inhibition of tubulin polymerisation as already shown. During cell cycle, the balance between tubulin dimers and microtubules plays a crucial role for successful mitosis.^[20]^ This supports the assumption that the cell cycle is also influenced by the inhibition of tubulin polymerisation as already shown. During cell cycle, the balance between tubulin dimers and microtubules plays a crucial role for successful mitosis.^[20]^

Obstruction of tubulin polymerisation also prevents the formation of kinetochore microtubules, which are supposed to separate the sister chromatids during anaphase, consequently leading to a G2/M‐phase arrest.^[21]^ A moderate increase of cells in the sub‐G1 phase was observed only for **1 a** and **1 d**, indicating that cell death is already occurring. To clarify whether an apoptotic mechanism was triggered, caspase‐3/7 activity was measured after compound treatment. No significant increase of this activity was observed, which supports the notion of an apoptosis‐independent cell death (Figure S2, Supporting Information). In line with the effect of centrosome de‐clustering, a mitotic catastrophe is the most likely mode of cell death, triggered by aberrant mitosis and leading to the formation of micronuclei and subsequent cell death.^[22]^ Cells that survive are more likely to get aneuploid through incorrect chromosome segregation causing chromosomal instability (CIN).^[23]^ CIN occurs in a variety of cancer types and is responsible for numerical changes in chromosomes.^[24]^ Alterations result in proliferative advantages but also increased susceptibility to the accumulation of chromosomal defects.^[25]^ Cell cycle regulating cyclin dependent kinases (CDKs) play a crucial role, as they control various cell cycle checkpoints and prevent uncontrolled cell division in normal cells.^[26]^ Furthermore, it has been shown that certain CDKs are overexpressed in tumor cells, which makes them an interesting target in cancer therapy.^[27]^ The CDKs 1, 2, 4, and 6 with their regulatory cyclin‐subunits A, B, D, and E are directly involved in cell cycle progression and essential for proliferation.^[28]^ Apart from CDK1, all other CDKs are redundant and are therefore not essential for successful cell division.^[29]^ The CDK1/Cyclin A2 ADP‐Glo assay system (Promega) was used to determine the inhibitory potential of **1 a**, **1 c** and **1 d** (Figure 3B). Although inferior to the positive control staurosporine (STA), **1 c** and **1 d** caused a significant decrease in CDK1‐activity at concentrations of 1 μm. In addition to their antimitotic properties, this is another mode of action that explains the G2/M‐arrest and provides a reason for the high selectivity for cancer over non‐malignant cells.

### Intracellular localization

**Figure 4:**
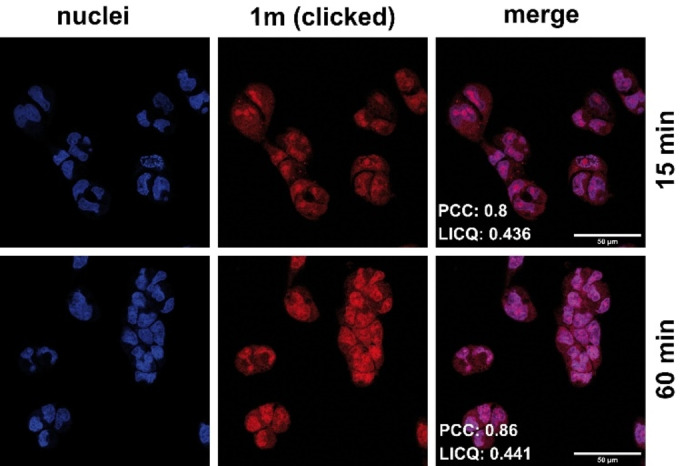
Localization of alkyne‐substituted compound **1 m** (50 μm) in 518 A2 melanoma cells after incubation for 15 and 60 min. The internalised compound was then fluorescently labelled using a copper(I)‐catalysed azide‐alkyne cycloaddition with 3‐azido‐7‐hydroxycoumarin (red, λ_ex_=404/λ_em_=477 nm). Colocalized nuclei were stained with Nuclear Green (blue, λ_ex_=503/λ_em_=526 nm). The experiment was carried out in triplicate. Magnification 630×. Pearson correlation coefficient (PCC) and Li's colocalization quotient (LICQ) were calculated for the merged images via imageJ (Coloc2 plugin).

The cellular localization of the ethynyl‐substituted derivative **1 m** in 518 A2 melanoma cells was investigated via copper catalysed azide‐alkyne click reactions using 3‐azido‐7‐hydroxycoumarin, CuSO_4_ and sodium ascorbate in bovine serum albumin (BSA) buffer (Figure 4).^[30]^

This fluorogenic method employs a bio‐orthogonal reaction proceeding under mild conditions and applicable to a variety of bioconjugations.^[31]^ Pearsons Correlation Coefficient (PCC) and Li's Intensity Correlation Quotient (LICQ) of cells co‐stained with DAPI (4′,6‐diamidino‐2‐phenylindole) were calculated using imageJ (Coloc2 plugin).^[32]^ High PCC and LICQ values of 0.86 (1 corresponds to a complete colocalization) and 0.441 (0.5 corresponds to a complete colocalization) after 60 min, indicated an accumulation of **1 m** mainly in the nuclei. Assuming that compounds **1 a**, **1 c**, and **1 d** accumulate in the nuclei, too, this finding is consistent with the observed CDK1 interference of the latter. Albeit not as strong as in the nucleus, a slight fluorescence occurs in the cytoplasm, that falls in line with the observed inhibition of tubulin polymerization, and correct spindle formation that take place predominantly in the cytoplasm.^[33]^

### Inhibition of tube‐formation by EA.hy926 cells

**Figure 5:**
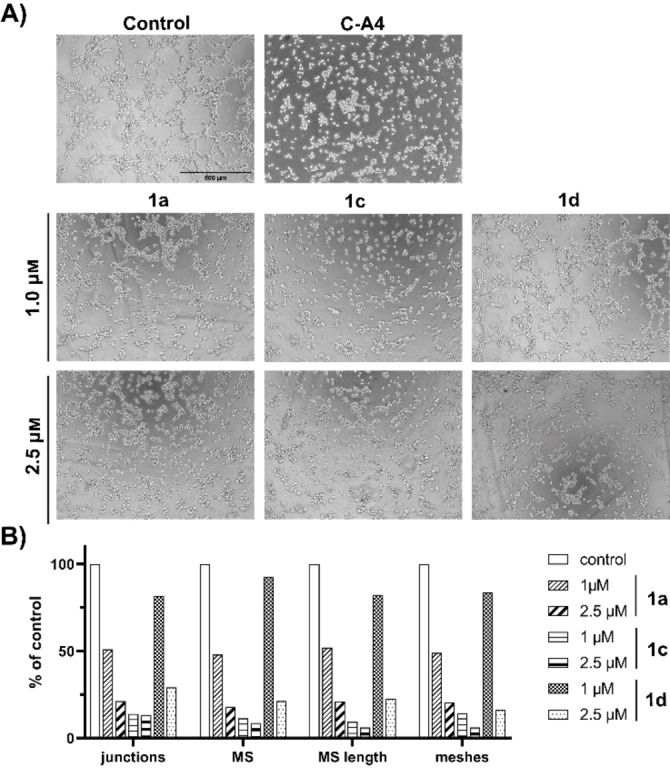
(A) Effect of compounds **1 a**, **1 c** and **1 d** (1 and 2.5 μm) on the ability of EA.hy926 endothelial hybrid cells to form vessel‐like structures on Matrigel® after 3 h. Negative controls were treated with vehicle (DMSO) and positive controls with C−A4 (100 nm). Images are representative of three independent experiments. The vitality (assessed by MTT‐assay) of treated cells was >80 % compared to controls set to 100 %. Magnification 100×. (B) Number of junctions, master segments (MS), MS length and meshes as representative parameters of tube‐formation assays as percent of control measured with imageJ (angiogenesis analysing plugin).^[41]^

Microtubule‐targeting agents (MTAs) do not only affect cell division and proliferation, but also the formation of new blood vessels.^[34]^ The recruitment of new blood vessels (angiogenesis) is essential for the growth of solid tumors, as it facilitates the supply of nutrients and oxygen.^[35]^ In addition, highly vascularized tumors have a considerably higher metastatic potential, due to a likelier intravasation of migrating cancer cells.[^36^, ^37^] For several MTAs such as paclitaxel, colchicine or C−A4, anti‐angiogenic effects were demonstrated in various studies, which primarily identified microtubule‐dependent processes like migration, cell‐adhesion or sprouting as targets.[^38^, ^39^] The *in vitro* tube formation assay was now used to determine the anti‐angiogenic properties of compounds **1 a**, **1 c**, and **1 d** (Figure 5A).^[40]^

This angiogenesis model is based on the ability of Ea.hy926 endothelial hybrid cells to form tubular and vessel‐like structures on a basement membrane matrix.^[42]^ For all three compounds, a concentration‐dependent effect was demonstrated. Compound **1 c** led to a complete inhibition of tube formation at concentrations as low as 1 μm, like the positive control C−A4. Compound **1 a** at 1 μm caused a decrease in cord‐like junctions but cells were still able to form small tubes and cell agglomerates. Compound **1 d** caused a significant reduction of vessel‐like structures only at 2.5 μM. The images were analysed with imageJ (angiogenesis‐analysing tool) and compared for the number of junctions, meshes, master segments (MS) and length of MS (Figure 5B). The vitality of the cells was determined by MTT assay to be higher than 80 % compared to negative controls (Table S3, Supporting Information).

### Antiangiogenic effects in zebrafish

**Figure 6:**
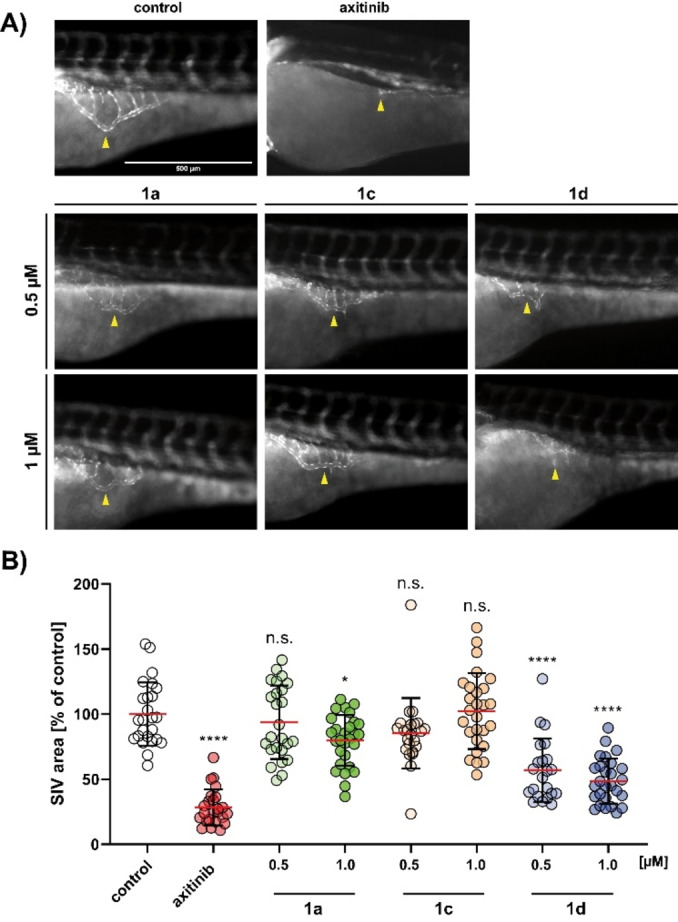
(A) Antiangiogenic effects of **1 a**, **1 c** and **1 d** (0.5 and 1 μm) on the subintestinal veins (SIV) of zebrafish embryos (24 hpf) after 48 h exposure. Negative controls were treated with vehicle DMSO and positive controls with axitinib (1 μm). Images are representative of at least 22 identically treated zebrafish. (B) Area of SIVs illustrated as the mean ±SD of each measurement was quantified using ImageJ. For **1 c** and **1 d**, concentrations above 1 μm were too toxic to be tested. The significance was given as *p<0.05; ****p<0.0001 against control, One‐way ANOVA, with Dunnett's multiple comparison test (GraphPad Prism 7).

As an *in vivo* study, the angiogenesis of zebrafish larvae 24 hours post fertilization (hpf) was observed by monitoring the development of the subintestinal veins (SIV) during treatment with **1 a**, **1 c** and **1 d** (0.5 and 1 μm).^[43]^ Transgenic *casper‐*zebrafish embryos (*fli1:EGFP*), lacking melanocytes and reflecting iridophores and expressing enhanced green fluorescent protein (EGFP) in their blood vessels, were used to analyse the anti‐angiogenic effects by fluorescence microscopy (Figure 6A).[^44^, ^45^]

Surprisingly, the most potent compound in tube formation inhibition, **1 c**, failed to show significant anti‐angiogenic effects *in vivo*. Concentrations above 1 μm of **1 c** and **1 d** were too toxic for the zebrafish larvae. **1 a** and **1 d**, however, showed a decrease in SIV‐area by 24 % (**1 a**, 1 μm) and 43 % (**1 d**, 1 μm) compared to untreated zebrafish (Figure 6B). In summary, while **1 d** had the strongest anti‐angiogenic effect, **1 a** was less toxic to vertebrates at concentrations above 1 μm.

## Conclusion

The evaluation of the fifteen 2‐amino‐4‐aryl‐5‐oxo‐4,5‐dihydropyrano[3,2‐*c*]chromene‐3‐carbonitriles **1 a**–**o** against a panel of tumor cell lines revealed that in particular, **1 a** (3,5‐dibromo‐4‐methoxyphenyl motif), **1 c** (3‐bromo‐4,5‐dimethoxyphenyl motif) and **1 d** (3,5‐diiodo‐4‐methoxyphenyl motif) with halogen‐substituted aryl residues proved highly active and selective against cancer cells of five different entities. Although not as potent as the remotely related vascular disrupting natural combretastatin‐A4, the compounds **1 a**, **1 c** and **1 d** inhibited tubulin polymerization and destabilized the microtubule cytoskeleton of cancer cells. Cancer cells treated with any one of these three compounds showed the phenomenon of centrosomal de‐clustering, normally appearing in cancer cells that cannot build a bipolar spindle apparatus, resulting in genome instability and subsequent cell death. Although many mechanisms of centrosome clustering are still unknown, it provides an effective and cancer‐specific target for future therapies.^[46]^ Intracellular localization of the ethynyl‐substituted derivative **1 m** via an alkyne‐azide click reaction to give a fluorescent triazole revealed its accumulation in the nucleus. This finding nicely matched the observed inhibition of CDK1/CyclinA2 and mitotic arrest by the **1 m**‐analogues **1 a**, **1 c** and **1 d**. Furthermore, concentration dependent anti‐angiogenic effects of these compounds were demonstrated *in vitro* by their inhibition of tube formation by EA.hy926 endothelial hybrid cells and *in vivo* by a significantly reduced development of subintestinal veins of zebrafish embryos treated with them. The development of new blood vessels, which is important for the growth and spread of tumors, can be inhibited by these substances, reducing cancer cell invasion and metastasis.[^36^, ^37^] In summary, the 3′,5′‐diiodo‐4′‐methoxy derivative **1 d** stood out as the most promising compound of this new series of 2‐amino‐4‐aryl‐5‐oxo‐4,5‐dihydropyrano[3,2‐*c*]chromene‐3‐carbonitriles with pleiotropic character. Based on its antiproliferative and antimetastatic properties, as well as its distinct selectivity for cancer cells and its rare centrosome declustering effect in cancer cells, **1 d** might be a good starting point for further optimization and investigation.

## Experimental Section

### General

All starting compounds were purchased from Aldrich, Alfa Aesar and TCI. The known compounds **1 n**–**q** were prepared according to literature procedures.[^10^, ^47^] The following instruments were applied for this study: melting points (uncorrected), Gallenkamp; IR spectra, Perkin‐Elmer Spectrum One FT‐IR spectrophotometer with ATR sampling unit; nuclear magnetic resonance spectra, BRUKER Avance 300 spectrometer; chemical shifts are given in parts per million (δ) downfield from tetramethylsilane as internal standard; mass spectra, Varian MAT 311 A (EI), UPLC/Orbitrap (ESI); microanalyses, Perkin‐Elmer 2400 CHN elemental analyzer. All tested compounds were >95 % pure by elemental analysis.

### Chemistry and Synthesis

#### 2‐Amino‐4‐(3‐bromo‐4,5‐dimethoxyphenyl)‐5‐oxo‐4,5‐dihydropyrano[3,2‐c]chromene‐3‐carbonitrile (1 a)

3‐Bromo‐4,5‐dimethoxybenzaldehyde (245 mg, 1.0 mmol) and malononitrile (70 mg, 1.0 mmol) were dissolved in MeCN (5 mL) and three drops of Et_3_N were added. The reaction mixture was stirred at room temperature for 30 min. 4‐Hydroxycoumarin (162 mg, 1.0 mmol) was added and the reaction mixture was stirred at room temperature for 16 h. The formed precipitate was collected, washed with MeCN and *n*‐hexane and dried in vacuum. Yield: 156 mg (0.34 mmol, 34 %); colorless solid of m.p. 237 °C; IR (ATR): *ν*_max_=3418, 3316, 3186, 2938, 2202, 1703, 1664, 1603, 1567, 1492, 1457, 1428, 1415, 1375, 1306, 1280, 1258, 1236, 1208, 1176, 1152, 1131, 1114, 1048, 998, 961, 904, 860, 833, 797, 769, 757, 784, 710, 676, 655, 629, 617, 577 cm^−1^; ^1^H NMR (300 MHz, [D_6_]DMSO): δ=3.71 (s, 3H), 3.80 (s, 3H), 4.48 (s, 1H), 6.98 (d, *J*=2.0 Hz, 1H), 7.02 (d, *J*=2.0 Hz, 1H), 7.4–7.5 (m, 4H), 7.7–7.8 (m, 1H), 7.89 (dd, *J*=7.9 Hz, 1.4 Hz, 1H); ^13^C NMR (75.5 MHz, [D_6_]DMSO): δ=36.5, 56.1, 57.4, 60.0, 103.1, 112.3, 113.1, 116.6, 119.1, 122.6, 123.0, 124.6, 132.9, 140.8, 144.7, 152.2, 153.2, 153.7, 158.0, 159.6; MS (70 eV): *m/z* (%): 456 (63) [*M*]^+^, 454 (66) [*M*]^+^, 425 (7), 423 (8), 390 (12), 388 (12), 375 (53), 359 (7), 309 (15), 239 (100), 121 (32), 66 (10); elemental analysis calcd (%) for C_21_H_15_BrN_2_O_5_: C 55.40, H 3.32, N 6.15; found: C 55.50, H 3.39, N 6.08.

#### 2‐Amino‐4‐(3‐iodo‐4,5‐dimethoxyphenyl)‐5‐oxo‐4,5‐dihydropyrano[3,2‐c]chromene‐3‐carbonitrile (1 b)

3‐Iodo‐4,5‐dimethoxybenzaldehyde (292 mg, 1.0 mmol) and malononitrile (70 mg, 1.0 mmol) were dissolved in MeCN (5 mL) and three drops of Et_3_N were added. The reaction mixture was stirred at room temperature for 30 min. 4‐Hydroxycoumarin (162 mg, 1.0 mmol) was added and the reaction mixture was stirred at room temperature for 16 h. The formed precipitate was collected, washed with MeCN and *n*‐hexane and dried in vacuum. Yield: 156 mg (0.34 mmol, 34 %); colorless solid of m.p. 237 °C; IR (ATR): *ν*_max_=3415, 3317, 3184, 2936, 2203, 1704, 1663, 1603, 1564, 1476, 1457, 1425, 1412, 1377, 1306, 1276, 1258, 1235, 1207, 1177, 1152, 1131, 1115, 1038, 998, 961, 903, 860, 820, 796, 767, 756, 721, 709, 675, 652, 628, 617 cm^−1^; ^1^H NMR (300 MHz, [D_6_]DMSO): δ=3.68 (s, 3H), 3.77 (s, 3H), 4.45 (s, 1H), 6.97 (s, 1H), 7.17 (s, 1H), 7.4–7.6 (m, 4H), 7.7–7.8 (m, 1H), 7.89 (d, *J*=7.9 Hz, 1H); ^13^C NMR (75.5 MHz, [D_6_]DMSO): δ=36.3, 56.0, 57.5, 59.7, 92.7, 103.3, 113.1, 116.6, 119.1, 122.6, 124.6, 128.6, 132.9, 141.5, 147.2, 152.1, 152.2, 153.6, 158.0, 159.6; MS (70 eV): *m/z* (%): 502 (63) [*M*]^+^, 436 (37), 309 (42), 239 (100), 121 (42); C_21_H_15_IN_2_O_5_: C 50.22, H 3.01, N 5.58; found: C 50.31, H 3.06, N 5.50.

#### 2‐Amino‐4‐(3,5‐dibromo‐4‐methoxyphenyl)‐5‐oxo‐4,5‐dihydropyrano[3,2‐c]chromene‐3‐carbonitrile (1 c)

3,5‐Dibromo‐4‐methoxybenzaldehyde (293 mg, 1.0 mmol) and malononitrile (70 mg, 1.0 mmol) were dissolved in MeCN (5 mL) and three drops of Et_3_N were added. The reaction mixture was stirred at room temperature for 30 min. The formed precipitate was redissolved by heating and 4‐hydroxycoumarin (162 mg, 1.0 mmol) was added. The reaction mixture was stirred at room temperature for 2 h. The formed precipitate was collected, washed with MeCN and *n*‐hexane and dried in vacuum. Yield: 240 mg (0.48 mmol, 48 %); colorless solid of m.p. 274–275 °C; IR (ATR): *ν*_max_=3388, 3322, 3256, 3215, 3194, 2944, 2923, 2857, 2822, 2197, 1714, 1667, 1639, 1605, 1547, 1495, 1466, 1456, 1420, 1398, 1381, 1324, 1307, 1276, 1257, 1207, 1176, 1115, 1050, 987, 957, 904, 872, 801, 772, 762, 749, 737, 719, 712, 676 cm^−1^; ^1^H NMR (300 MHz, [D_6_]DMSO): δ=3.78 (s, 3H), 4.53 (s, 1H), 7.4–7.5 (m, 2H), 7.51 (s, 2H), 7.60 (s, 2H), 7.7–7.8 (m, 1H), 7.8–7.9 (m, 1H); ^13^C NMR (75.5 MHz, [D_6_]DMSO): δ=35.9, 57.0, 60.4, 102.6, 113.1, 116.6, 117.4, 119.0, 122.7, 124.6, 132.1, 133.0, 142.6, 152.3, 154.0, 158.0, 159.7; MS (70 eV): *m/z* (%): 506 (9) [*M*]^+^, 504 (18) [*M*]^+^, 502 (9) [*M*]^+^, 438 (9), 425 (15), 423 (16), 359 (12), 357 (11), 239 (100), 121 (23); C_20_H_12_Br_2_N_2_O_4_: C 47.65, H 2.40, N 5.56; found: C 47.72, H 2.45, N 5.49.

#### 2‐Amino‐4‐(3,5‐diiodo‐4‐methoxyphenyl)‐5‐oxo‐4,5‐dihydropyrano[3,2‐c]chromene‐3‐carbonitrile (1 d)

3,5‐Diiodo‐4‐methoxybenzaldehyde (388 mg, 1.0 mmol) and malononitrile (70 mg, 1.0 mmol) were dissolved in MeCN (3 mL) and three drops of Et_3_N were added. The reaction mixture was stirred at room temperature for 30 min. 4‐Hydroxycoumarin (162 mg, 1.0 mmol) was added, the reaction mixture was stirred at room temperature for 1 h. The formed precipitate was collected, washed with MeCN/H_2_O, and dried in vacuum. Yield: 322 mg (0.54 mmol, 54 %); colorless solid of m.p. 277–278 °C; IR (ATR): *ν*_max_=3655, 3322, 3288, 3254, 3209, 2177, 2932, 2197, 1712, 1673, 1637, 1607, 1538, 1492, 1457, 1412, 1400, 1376, 1327, 1310, 1284, 1272, 1251, 1211, 1171, 1112, 1059, 995, 958, 907, 800, 768, 746, 736, 710, 699 cm^−1^; ^1^H NMR (300 MHz, [D_6_]DMSO): δ=3.73 (s, 3H), 4.47 (s, 1H), 7.4–7.5 (m, 4H), 7.7–7.8 (m, 3H), 7.89 (d, *J*=7.9 Hz, 1H); ^13^C NMR (75.5 MHz, [D_6_]DMSO): δ=35.3, 57.1, 60.2, 91.4, 102.8, 113.1, 116.6, 119.0, 122.6, 124.6, 133.0, 138.7, 143.1, 152.2, 153.8, 157.4, 158.0, 159.6; MS (70 eV): *m/z* (%): 598 (20) [*M*]^+^, 532 (15), 405 (31), 239 (100), 121 (23); C_20_H_12_I_2_N_2_O_4_: C 40.16, H 2.02, N 4.68; found: C 40.28, H 2.08, N 4.62.

#### 2‐Amino‐4‐(3,4‐difluorophenyl)‐5‐oxo‐4,5‐dihydropyrano[3,2‐c]chromene‐3‐carbonitrile (1 e)

3,4‐Difluorobenzaldehyde (142 mg, 1.0 mmol) and malononitrile (70 mg, 1.0 mmol) were dissolved in MeCN (5 mL) and three drops of Et_3_N were added. The reaction mixture was stirred at room temperature for 30 min. The formed precipitate was redissolved by heating and 4‐hydroxycoumarin (162 mg, 1.0 mmol) was added. The reaction mixture was stirred at room temperature for 16 h. The formed precipitate was collected, washed with MeCN and *n*‐hexane and dried in vacuum. Yield: 180 mg (0.51 mmol, 51 %); colorless solid of m.p. 248–249 °C; IR (ATR) *ν*_max_=3366, 3323, 3301, 3261, 3193, 2942, 2200, 1716, 1673, 1641, 1606, 1578, 1519, 1493, 1456, 1436, 1411, 1376, 1326, 1315, 1288, 1274, 1257, 1208, 1171, 1137, 1112, 1056, 1023, 960, 932, 922, 905, 871, 838, 823, 808, 778, 763, 753, 745, 722, 710, 698, 677, 648, 626, 615, 599, 588, 576 cm^−1^; ^1^H NMR (300 MHz, [D_6_]DMSO): δ=4.53 (s, 1H), 7.1–7.2 (m, 1H), 7.3–7.5 (m, 6H), 7.7–7.8 (m, 1H), 7.89 (dd, *J*=7.9 Hz, 1.4 Hz, 1H); ^13^C NMR (75.5 MHz, [D_6_]DMSO): δ=36.3, 57.3, 103.0, 113.0, 116.5, 116.7, 116.9, 117.2, 117.4, 119.0, 122.6, 124.6, 133.0, 141.1, 147.6, 150.2, 152.2, 153.7, 157.9, 159.6; MS (70 eV): *m/z* (%): 352 (34) [*M*]^+^, 239 (100), 121 (31); C_19_H_10_F_2_N_2_O_3_: C 64.78, H 2.86, N 7.95; found: C 64.84, H 2.90, N 7.89.

#### 2‐Amino‐4‐(3,5‐difluorophenyl)‐5‐oxo‐4,5‐dihydropyrano[3,2‐c]chromene‐3‐carbonitrile (1 f)

3,5‐Difluorobenzaldehyde (142 mg, 1.0 mmol) and malononitrile (70 mg, 1.0 mmol) were dissolved in MeCN (5 mL) and three drops of Et_3_N were added. The reaction mixture was stirred at room temperature for 30 min. The formed precipitate was redissolved by heating and 4‐hydroxycoumarin (162 mg, 1.0 mmol) was added. The reaction mixture was stirred at room temperature for 16 h. The formed precipitate was collected, washed with MeCN and *n*‐hexane and dried in vacuum. Yield: 95 mg (0.27 mmol, 27 %); colorless solid of m.p. 238 °C; IR (ATR): *ν*_max_=3406, 3322, 3256, 3218, 3193, 3086, 3055, 2195, 1694, 1664, 1622, 1606, 1594, 1495, 1456, 1445, 1416, 1382, 1305, 1276, 1253, 1205, 1179, 1154, 1124, 1115, 1063, 1022, 993, 970, 945, 905, 856, 796, 782, 766, 759, 720, 688, 672 cm^−1^; ^1^H NMR (300 MHz, CDCl_3_/ D_6_]DMSO): δ=4.50 (s, 1H), 6.46 (s, 2H), 6.6–6.7 (m, 1H), 6.8–6.9 (m, 2H), 7.3–7.4 (m, 2H), 7.5–7.6 (m, 1H), 7.83 (dd, *J*=9.5 Hz, 1.6 Hz, 1H); ^13^C NMR (75.5 MHz, D_6_]DMSO): δ=36.4, 58.2, 102.0, 102.4, 102.7, 103.0, 110.2, 110.5, 112.4, 116.2, 118.2, 122.3, 124.1, 132.4, 146.0, 152.1, 153.6, 158.0, 159.5, 160.6, 160.8, 164.1; MS (70 eV): *m/z* (%): 352 (42) [*M*]^+^, 240 (23), 239 (100), 121 (23); C_19_H_10_F_2_N_2_O_3_: C 64.78, H 2.86, N 7.95; found: C 64.82, H 2.91, N 7.90.

#### 2‐Amino‐4‐(3,4,5‐trifluorophenyl)‐5‐oxo‐4,5‐dihydropyrano[3,2‐c]chromene‐3‐carbonitrile (1 g)

3,4,5‐Trifluorobenzaldehyde (160 mg, 1.0 mmol) and malononitrile (70 mg, 1.0 mmol) were dissolved in MeCN (3 mL) and three drops of Et_3_N were added. The reaction mixture was stirred at room temperature for 30 min. 4‐Hydroxycoumarin (162 mg, 1.0 mmol) was added, the reaction mixture was stirred at room temperature for 1 h. The formed precipitate was collected, washed with MeCN/H_2_O, and dried in vacuum. Yield: 145 mg (0.39 mmol, 39 %); colorless solid of m.p. 236–237 °C; IR (ATR): *ν*_max_=3402, 3319, 3257, 3212, 3191, 2198, 1708, 1665, 1629, 1604, 1528, 1495, 1448, 1414, 1380, 1342, 1307, 1275, 1254, 1239, 1204, 1178, 1151, 1114, 1056, 1040, 972, 953, 905, 865, 809, 796, 780, 757, 715, 706, 684, 662 cm^−1^; ^1^H NMR (300 MHz, [D_6_]DMSO): δ=4.57 (s, 1H), 7.3–7.4 (m, 2H), 7.5–7.6 (m, 4H), 7.7–7.8 (m, 1H), 7.89 (d, *J*=7.9 Hz, 1H); ^13^C NMR (75.5 MHz, [D_6_]DMSO): δ=36.4, 56.8, 102.3, 112.4, 112.7, 113.1, 116.5, 118.8, 122.7, 124.6, 133.0, 140.7, 148.5, 152.3, 154.1, 158.0, 159.6; MS (70 eV): *m/z* (%): 370 (28) [*M*]^+^, 239 (100), 121 (44), 92 (23); C_19_H_9_F_3_N_2_O_3_: C 61.63, H 2.45, N 7.57; found: C 61.71, H 2.50, N 7.51.

#### 2‐Amino‐4‐(3,4‐dichlorophenyl)‐5‐oxo‐4,5‐dihydropyrano[3,2‐c]chromene‐3‐carbonitrile (1 h)

3,4‐Dichlorobenzaldehyde (175 mg, 1.0 mmol) and malononitrile (70 mg, 1.0 mmol) were dissolved in MeCN (3 mL) and three drops of Et_3_N were added. The reaction mixture was stirred at room temperature for 30 min. 4‐Hydroxycoumarin (162 mg, 1.0 mmol) was added, the reaction mixture was stirred at room temperature for 1 h. The formed precipitate was collected, washed with MeCN/H_2_O, and dried in vacuum. Yield: 190 mg (0.49 mmol, 49 %); colorless solid of m.p. 244–245 °C; IR (ATR): *ν*_max_=3394, 3320, 3250, 3209, 3192, 2206, 1694, 1666, 1604, 1496, 1468, 1456, 1399, 1378, 1312, 1275, 1254, 1208, 1177, 1147, 1114, 1061, 1027, 958, 906, 838, 780, 761, 730, 699, 675 cm^−1^; ^1^H NMR (300 MHz, [D_6_]DMSO): δ=4.55 (s, 1H), 7.3–7.4 (m, 1H), 7.4–7.6 (m, 6H), 7.7–7.8 (m, 1H), 7.89 (d, *J*=7.8 Hz, 1H); ^13^C NMR (75.5 MHz, [D_6_]DMSO): δ=36.2, 57.0, 102.8, 113.0, 116.6, 118.9, 122.6, 124.6, 128.3, 129.7, 129.9, 130.6, 131.0, 133.0, 144.4, 152.3, 153.9, 158.0, 159.6; MS (70 eV): *m/z* (%): 386 (11) [*M*]^+^, 384 (16) [*M*]^+^, 239 (100), 121 (22); C_19_H_10_Cl_2_N_2_O_3_: C 59.24, H 2.62, N 7.27; found: C 59.32, H 2.68, N 7.22.

#### 2‐Amino‐4‐(4‐methylthiophenyl)‐5‐oxo‐4,5‐dihydropyrano[3,2‐c]chromene‐3‐carbonitrile (1 i)

4‐Methylsulfanylbenzaldehyde (152 mg, 1.0 mmol) and malononitrile (70 mg, 1.0 mmol) were dissolved in MeCN (5 mL) and three drops of Et_3_N were added. The reaction mixture was stirred at room temperature for 30 min. The formed precipitate was redissolved by heating and 4‐hydroxycoumarin (162 mg, 1.0 mmol) was added. The reaction mixture was stirred at room temperature for 16 h. The formed precipitate was collected, washed with MeCN and *n*‐hexane and dried in vacuum. Yield: 150 mg (0.41 mmol, 41 %); colorless solid of m.p. 235 °C; IR (ATR): *ν*_max_=3388, 3321, 3253, 3192, 2917, 2193, 1705, 1667, 1605, 1493, 1456, 1435, 1407, 1378, 1328, 1305, 1273, 1253, 1206, 1175, 1112, 1090, 1047, 1014, 957, 903, 834, 773, 755, 727, 700, 676, 646, 618, 578, 558 cm^−1^; ^1^H NMR (300 MHz, [D_6_]DMSO): δ=2.44 (s, 3H), 4.42 (s, 1H), 7.1–7.2 (m, 4H), 7.4–7.5 (m, 4H), 7.7–7.8 (m, 1H), 7.90 (dd, *J*=7.9 Hz, 1.4 Hz, 1H); ^13^C NMR (75.5 MHz, [D_6_]DMSO): δ=14.7, 36.5, 57.8, 103.9, 113.0, 116.6, 119.2, 122.5, 124.7, 126.0, 128.3, 132.9, 136.8, 140.0, 152.1, 153.3, 157.9, 159.5; MS (70 eV): *m/z* (%): 362 (96) [M]^+^, 315 (70). 296 (25), 249 (42), 239 (100), 121 (24); C_20_H_14_N_2_O_3_S: C 66.29, H 3.89, N 7.73; found: C 66.35, H 3.96, N 7.77.

#### 2‐Amino‐4‐(3‐pentafluorothiophenyl)‐5‐oxo‐4,5‐dihydropyrano[3,2‐c]chromene‐3‐carbonitrile (1 j)

3‐Pentafluorothiobenzaldehyde (232 mg, 1.0 mmol) and malononitrile (70 mg, 1.0 mmol) were dissolved in MeCN (5 mL) and three drops of Et_3_N were added. The reaction mixture was stirred at room temperature for 30 min. The formed precipitate was redissolved by heating and 4‐hydroxycoumarin (162 mg, 1.0 mmol) was added. The reaction mixture was stirred at room temperature for 16 h. The formed precipitate was collected, washed with MeCN and *n*‐hexane and dried in vacuum. Yield: 270 mg (0.61 mmol, 61 %); colorless solid of m.p. 285 °C; IR (ATR): *ν*_max_=3416, 3326, 3256, 3222, 3194, 3117, 3076, 2200, 1706, 1668, 1636, 1604, 1495, 1484, 1456, 1435, 1411, 1380, 1331, 1305, 1275, 1260, 1206, 1176, 1114, 1053, 956, 907, 881, 851, 835, 817, 789, 771, 761, 747, 725, 710, 687 cm^−1^; ^1^H NMR (300 MHz, [D_6_]DMSO): δ=4.70 (s, 1H), 7.4–7.6 (m, 6H), 7.7–7.9 (m, 3H), 7.91 (dd, *J*=7.9 Hz, 1.4 Hz, 1H); ^13^C NMR (75.5 MHz, [D_6_]DMSO): δ=36.8, 57.1, 102.9, 112.9, 116.6, 118.9, 122.6, 124.7, 125.1, 129.8, 131.7, 133.1, 145.0, 152.3, 152.6, 153.9, 158.1, 159.6; MS (70 eV): *m/z* (%): 442 (18) [*M*]^+^, 314 (8), 239 (100), 121 (17); C_19_H_11_F_5_N_2_O_3_S: C 51.59, H 2.51, N 6.33; found: C 51.67, H 2.56, N 6.30.

#### 2‐Amino‐4‐(3‐nitro‐4‐methoxyphenyl)‐5‐oxo‐4,5‐dihydropyrano[3,2‐c]chromene‐3‐carbonitrile (1 k)

4‐Methoxy‐3‐nitrobenzaldehyde (181 mg, 1.0 mmol) and malononitrile (70 mg, 1.0 mmol) were dissolved in MeCN (5 mL) and three drops of Et_3_N were added. The reaction mixture was stirred at room temperature for 30 min. The formed precipitate was redissolved by heating and 4‐hydroxycoumarin (162 mg, 1.0 mmol) was added. The reaction mixture was stirred at room temperature for 16 h. The formed precipitate was collected, washed with MeCN and *n*‐hexane and dried in vacuum. Yield: 180 mg (0.46 mmol, 46 %); colorless solid of m.p. 246–247 °C; IR (ATR): *ν*_max_=3368, 3329, 3298, 3284, 3249, 3211, 3187, 3055, 2947, 2846, 2196, 1716, 1698, 1671, 1619, 1604, 1578, 1526, 1497, 1458, 1411, 1378, 1356, 1321, 1308, 1281, 1251, 1210, 1181, 1114, 1087, 1063, 1017, 963, 906, 845, 824, 763, 702, 672 cm^−1^; ^1^H NMR (300 MHz, [D_6_]DMSO): δ=3.90 (s, 3H), 4.57 (s, 1H), 7.30 (d, *J*=8.8 Hz, 1H), 7.4–7.5 (m, 4H), 7.6–7.8 (m, 2H), 7.79 (d, *J*=2.3 Hz, 1H), 7.9–8.0 (m, 1H); ^13^C NMR (75.5 MHz, [D_6_]DMSO): δ=35.9, 56.7, 57.2, 103.0, 113.1, 114.2, 116.6, 119.1, 122.6, 123.9, 124.6, 133.0, 133.8, 135.8, 139.2, 150.9, 152.2, 153.7, 158.0, 159.6; MS (70 eV): *m/z* (%): 391 (22) [*M*]^+^, 374 (20), 325 (20), 239 (100), 121 (27); C_20_H_13_N_3_O_6_: C 61.38, H 3.35, N 10.74; found: C 61.46, H 3.41, N 10.66.

#### 2‐Amino‐4‐(3‐cyanophenyl)‐5‐oxo‐4,5‐dihydropyrano[3,2‐c]‐ chromene‐3‐carbonitrile (1 l)

3‐Cyanobenzaldehyde (131 mg, 1.0 mmol) and malononitrile (70 mg, 1.0 mmol) were dissolved in MeCN (3 mL) and three drops of Et_3_N were added. The reaction mixture was stirred at room temperature for 30 min. The formed precipitate was redissolved by heating and 4‐hydroxycoumarin (162 mg, 1.0 mmol) was added. The reaction mixture was stirred at room temperature for 1 h. The formed precipitate was collected, washed with MeCN and *n*‐hexane and dried in vacuum. Yield: 197 mg (0.58 mmol, 58 %); colorless solid of m.p. 278 °C; IR (ATR): *ν*_max_=3400, 3322, 3291, 3253, 3215, 3194, 3076, 2233, 2201, 1722, 1675, 1638, 1602, 1495, 1456, 1435, 1417, 1376, 1327, 1302, 1273, 1255, 1213, 1196, 1171, 1112, 1098, 1056, 1012, 957, 928, 901, 876, 817, 789, 779, 762, 744, 703 cm^−1^; ^1^H NMR (300 MHz, [D_6_]DMSO): δ=4.59 (s, 1H), 7.4–7.6 (m, 5H), 7.6–7.8 (m, 3H),7.8–7.9 (m, 2H); ^13^C NMR (75.5 MHz, [D_6_]DMSO): δ=36.6, 57.1, 102.7, 111.4, 113.1, 116.6, 118.7, 119.0, 122.6, 124.6, 129.7, 131.0, 131.5, 132.9, 133.0, 144.9, 152.3, 154.0, 158.0, 159.6; MS (70 eV): *m/z* (%): 341 (33) [*M*]^+^, 275 (19), 274 (24), 240 (22), 239 (100), 121 (26); C_20_H_11_N_3_O_3_: C 70.38, H 3.25, N 12.31; found: C 70.42, H 3.30, N 12.27.

#### 2‐Amino‐4‐(4‐ethynylphenyl)‐5‐oxo‐4,5‐dihydropyrano[3,2‐c]‐ chromene‐3‐carbonitrile (1 m)

3‐Ethynylbenzaldehyde (130 mg, 1.0 mmol) and malononitrile (70 mg, 1.0 mmol) were dissolved in MeCN (5 mL) and three drops of Et_3_N were added. The reaction mixture was stirred at room temperature for 30 min. The formed precipitate was redissolved by heating and 4‐hydroxycoumarin (162 mg, 1.0 mmol) was added. The reaction mixture was stirred at room temperature for 1 h. The formed precipitate was collected, washed with MeCN and *n*‐hexane and dried in vacuum. Yield: 85 mg (0.25 mmol, 25 %); off‐white solid of m.p. >395 °C; IR (ATR): *ν*_max_=3390, 3322, 3285, 3212, 2196, 1709, 1669, 1639, 1605, 1498, 1456, 1417, 1381, 1328, 1307, 1275, 1257, 1210, 1177, 1115, 1052, 1018, 958, 904, 852, 833, 787, 773, 740, 661 cm^−1^; ^1^H NMR (300 MHz, [D_6_]DMSO): δ=4.15 (s, 1H), 4.49 (s, 1H), 7.28 (dd, *J*=8.3 Hz, 1.9 Hz, 2H), 7.4–7.5 (m, 6H), 7.7–7.8 (m, 1H), 7.9–8.0 (m, 1H); ^13^C NMR (75.5 MHz, [D_6_]DMSO): δ=36.8, 57.4, 80.8, 83.3, 103.5, 112.9, 116.6, 119.1, 120.5, 122.5, 124.7, 128.1, 131.9, 133.0, 144.2, 152.2, 153.6, 158.0, 159.6; MS (70 eV): *m/z* (%): 340 (52) [*M*]^+^, 273 (24), 239 (100), 121 (37); C_19_H_10_F_2_N_2_O_3_: C 64.78, H 2.86, N 7.95; found: C 64.84, H 2.90, N 7.89; C_21_H_12_N_2_O_3_: C 74.11, H 3.55, N 8.23; found: C 74.22, H 3.48, N 8.19.

### Cell lines and culture conditions

518 A2 melanoma (Department of Radiotherapy, Medical University of Vienna, Austria),^[48]^ KB‐V1^Vbl^ (ACC‐149) multidrug‐resistant (MDR) cervix carcinoma, U‐87 glioblastoma, MCF‐7 (ACC‐115) breast carcinoma, HT‐29 (ACC‐299), HCT‐116 (ACC‐581) and HCT‐116p53−/− (p53 knockout mutant) colon carcinoma, EA.hy926 (ATCC® CRL‐2922^TM^) endothelial hybrid cells, and HDFa (ATCC® PCS‐201‐012^TM^) human dermal fibroblasts were cultivated in Dulbecco's Modified Eagle Medium (DMEM) supplemented with 10 % fetal bovine serum (20 % for HDFa cells), and 1 % antibiotic‐antimycotic at 37 °C, 5 % CO_2_, and 95 % humidity. To keep KB‐V1^Vbl^ cells resistant, 340 nM vinblastine was added to the cell culture medium 24 h after every passage. Cells were grown at 5 % CO_2_, and 95 % humidity. Only mycoplasma‐free cell cultures were used.

### Cell viability assay (MTT assay)

MTT assays were carried out for cytotoxicity evaluation of compounds **1 a**–**q**. The cancer and hybrid cells (5×10^4^ cells/mL, 100 μL/well), as well as HDFa cells (1×10^5^ cells/mL, 100 μL/well) were grown in 96‐well plates for 24 h under cell culture conditions. Then, they were treated with various concentrations (100 μm–0.5 nm) of compounds **1 a**–**q**, or vehicle (DMSO) for another 72 h. After the addition of 12.5 μL of a 0.5 % MTT solution in PBS the cells were incubated for 2 h at 37 °C so that the water‐soluble MTT could be converted to formazan crystals. The plates were centrifuged (300 g, 5 min, 4 °C), the medium was withdrawn, and the formazan dissolved in 25 μL of DMSO containing 10 % SDS and 0.6 % acetic acid for at least 1 h at 37 °C. The absorbance of formazan (λ=570 nm), and background (λ=630 nm) was measured with a microplate reader (Tecan infinite F200). The IC_50_ values were derived from dose‐inhibition curves as the means ±SD of four independent experiments with respect to vehicle treated control cells set to 100 % (GraphPad Prism). The cytotoxic selectivity was determined by calculating the selectivity index (SI) according to following equation: SI=IC_50_ (nonmalignant HDFa cells)/IC_50_ (average cancer cell lines).

### Tubulin polymerisation

In a black 96‐well half‐area clear bottom plate, 50 μL of Brinkley's Buffer 80 (BRB80: 400 mM PIPES, 5 mM MgCl_2_, 5 mM EGTA, pH=6.8) containing 20 % glycerol and 2 mM GTP was pipetted. Test compounds were added to the wells to reach a concentration of 5 μm. To start the polymerization reaction 50 μL porcine brain tubulin (10 mg/mL in BRB80) was added and the plate was immediately placed in the microplate reader (Tecan infinite F200). The optical density was measured at 37 °C by recording the absorption at 340 nm for 100 min. Data are representative as the means ±SD of at least two independent experiments.

### Immunofluorescence staining of tubulin cytoskeleton

518 A2 melanoma cells (1×10^5^ cells/mL, 0.5 mL/well) were seeded on coverslips in 24‐well cell culture plates and incubated for 24 h under cell culture conditions (37 °C, 5 % CO_2_ and 95 % humidity). After treatment with different concentrations of test compounds **1 a**, **1 c** and **1 d** (1 and 2.5 μm) or the vehicle DMSO, the cells were incubated for another 24 h (37 °C, 5 % CO_2_, 95 % humidity). The cells were washed with cytoskeletal buffer (100 mm PIPES, 3 mM MgCl_2_, 138 mM KCl, 2 mM EGTA, 300 mM sucrose, pH 6.8), fixed and permeabilized in 3.7 % formaldehyde and 0.2 % Triton X‐100 in cytoskeletal buffer for 5 min at rt. As additional fixation step, the cells were incubated with ice‐cold EtOH for 10 s and rehydrated in PBS. After blocking with 1 % BSA in PBS for 30 min the cells were treated for 2 h with a primary antibody against alpha‐tubulin (anti alpha‐tubulin, mouse monoclonal antibody), washed two times with PBS, followed by 1 h incubation with a secondary antibody AlexaFluor®‐546 conjugate (goat anti‐mouse IgG‐AF‐546, Invitrogen). Actin and nuclei staining was done with Phallodin‐iFluor^TM^ 488 Conjugate (AAT Bioquest) and DAPI (1 μg/mL) for 1 h in the dark. Finally, the cells were washed three times with PBS and the coverslips were embedded in ProLong^TM^ Glass Antifade Mountant. Nuclei, actin filaments and microtubules were documented by fluorescence microscopy (Zeiss Imager A1 AX10, 100× and 630× magnification) and edited with imageJ. The ratio of bipolar and multipolar spindle apparatus to the total cell count was determined with the cell count tool (imageJ).

### Cell cycle analysis

518 A2 melanoma cells (1×10^5^ cells/mL, 3 mL/well) were seeded in 6‐well cell culture plates for 24 h under cell culture conditions (37 °C, 5 % CO_2_, 95 % humidity). The treatment with 1 μm each of compounds **1 a**, **1 c**, **1 d**, vehicle (DMSO) or positive control C−A4 (25 nm) was carried out for another 12 h. Cells were fixed in 70 % EtOH after trypsinization and centrifugation (5 min, 300×g, 4 °C) for at least 24 h at 4 °C. Before flow‐cytometric measurement with a Beckmann Coulter Cytomics FC500 flow cytometer (λ_em_=570 nm, λ_ex_=488 nm laser source) the cells were washed with PBS and stained with propidium iodide solution (50 μg/mL PI, 0.1 % sodium citrate, 50 μg/mL RNAse A in PBS). The DNA content of at least 10,000 single cells was measured and the ratio of the cell cycle phases (sub‐G1, G1, S, G2/M) were determined by CXP software (Beckman Coulter).

### CDK1/CyclinA2 activity assay

The CDK1/CyclinA2 kinase enzyme system (Promega) was used to profile the effect of substance **1 a**, **1 c**, **1 d**, staurosporine, or vehicle DMSO on kinase activity.^[49]^ The released ADP was quantified by ADP‐Glo^TM^ Assay (Promega).^[50]^ Reactions were carried out in white 96‐well plates with 2 ng of CDK1/CyclinA2 protein complex, 5 μg histone H1 substrate, and test compounds in reaction buffer (40 mM Tris‐HCl, 20 mM MgCl_2_, 0.1 mg/mL BSA, pH 7.5). The kinase reaction was initiated by adding 5 μL of ATP solution (250 μm), bringing the final volume up to 25 μL. The reaction was terminated after 15 min at 30 °C and remaining ATP was depleted by addition 25 μL of ADP‐Glo reagent for 40 min at rt. 50 μL of kinase detection reagent was added for another 30 min of incubation and followed by luminescence measurement with a FLUOstar microplate reader (Omega). Each concentration was measured in duplicate and solvent controls were set to 100 %. Significant decrease in CDK1/Cyclin A2 activity compared to vehicle control was determined using a *t*‐test; *p<0.05; **p<0.01; ***p<0.001; ****p<0.0001, One‐way ANOVA with Dunnett's multiple comparison test (GraphPad Prism 7).

### Colocalization via intracellular click‐reaction

518 A2 melanoma cells (1×10^5^ cells/mL, 0.5 mL/well) were grown on coverslips in 24‐well cell culture plates for 24 h under cell culture conditions (37 °C, 5 % CO_2_, 95 % humidity). Then the cells were incubated with 50 μm **1 m** (solution in 0.2 % Tween 80) for 15, 30 and 60 min under cell culture conditions. After washing with PBS, the cells were fixed (3.7 % formaldehyde in PBS) for 10 min and again washed with PBS. The “click‐reagents” (2 mM CuSO_4_, 5 mM sodium ascorbate, 0.1 mM 3‐azido‐7‐hydroxycoumarin, 1 % BSA in PBS) were incubated before 200 μL was pipetted to the cells and incubated in the dark for 30 min. Nuclei were counterstained with Nuclear Green (1 : 1000, 50 μg/mL RNase, 1 % BSA in PBS). The cells were washed once more with PBS and ddH_2_O before the coverslips were embedded in Roti®Mount FluorCare and documented using a Leica TCS SP5 confocal microscope (630× magnification). Pearson correlation coefficient (PC) and Li's colocalization quotient (LICQ) were calculated for the merged images via imageJ (Coloc2).

### EA.hy926 tube formation assay

Ibidi μ‐Slides were coated with the basement membrane‐like matrix Matrigel® (Corning). EA.hy926 endothelial hybrid cells were cultivated for 24 h in EndoPrime low serum (Capricorn) endothelial medium and seeded (50 μL/well, 3×10^5^ cells/mL) on Matrigel®. The cells were treated with **1 a**, **1 c**, **1 d** (1 and 2.5 μm) or solvent (DMSO) for 4 h under cell culture conditions (37 °C, 5 % CO_2_, and 95 % humidity), until tubular structures had formed in the control wells. Anti‐angiogenic effects were documented via light microscopy (Zeiss Axiovert 135, 100× magnification). The measurements were carried out in triplicate. Cell vitality was reviewed via MTT assay (higher than 75 % with DMSO treated cells set to 100 %) as described above (2.4.). The images were analysed with imageJ (angiogenesis‐analysing tool) and compared for the number of junctions, meshes, master segments (MS) and length of MS to quantify the tube formation process.^[39]^

### Zebrafish angiogenesis assay

Transgenic zebrafish of the strain *Tg(fli1:EGFP)* and *casper* mutant background were raised under standard conditions at about 28 °C.[^44^, ^51^] The eggs were cultivated in E3 medium (5 mM NaCl, 0.17 mM KCl, 0.33 mM CaCl_2_, 0.33 mM MgSO_4_, 0.01 % methylene blue, pH 7.2) for 24 h, manually dechorionated and distributed in 6‐well plates with 5 mL E3 medium each. The embryos were treated with **1 a**, **1 c** and **1 d** (0.5, 1 and 2.5 μm), axitinib (1 μm), or solvent DMSO for 48 h. The SIV (subintestinal vein) area was used to determine the vascular development and angiogenesis respectively, and was documented by fluorescence microscopy (λ_ex_=488 nm, λ_em_=509 nm; Leica MZ10F with Zeiss AxioCam Mrc and Mrc‐ZEN pro 2012 software). SIV areas were quantified with imageJ of at least 22 identically treated zebrafish larvae per concentration as means ±SD with solvent control set to 100 %. Significance in SIV decrease compared to vehicle control was determined using a *t*‐test; *p<0.05; **p<0.01; ***p<0.001; ****p<0.0001, One‐way ANOVA with Dunnett's multiple comparison test (GraphPad Prism 7).

## Conflict of interest

The authors declare no conflict of interest.

1

## Supporting information

As a service to our authors and readers, this journal provides supporting information supplied by the authors. Such materials are peer reviewed and may be re‐organized for online delivery, but are not copy‐edited or typeset. Technical support issues arising from supporting information (other than missing files) should be addressed to the authors.

Supporting Information

## Data Availability

The data that support the findings of this study are available from the corresponding author upon reasonable request.
