# Supplementary material for: 2‐Amino‐4‐aryl‐5‐oxo‐4,5‐dihydropyrano[3,2‐c]chromene‐3‐carbonitriles with Microtubule‐Disruptive, Centrosome‐Declustering, and Antiangiogenic Effects in vitro and in vivo
Source: ChemMedChem. 2022 Mar 16;17(10):e202200064. doi: 10.1002/cmdc.202200064 (PMC9311119; doi:10.1002/cmdc.202200064)
Supplement: Supplementary file 1 — Supporting Information [file CMDC-17-0-s001.pdf]

ChemMedChem

Supporting Information

2-Amino-4-aryl-5-oxo-4,5-dihydropyrano[3,2-*c*]chromene-3-carbonitriles with Microtubule-Disruptive, Centrosome-Declustering, and Antiangiogenic Effects *in vitro* and *in vivo*

Leonhard H. F. Köhler, Sebastian Reich, Gerrit Begemann, Rainer Schobert, and Bernhard Biersack*

Table of Contents

Experimental section 2

NMR Spectra 2

Results 33

Selectivity Index 33

Effects on the cell cycle 33

Caspase-3/7 activity 34

Tube-Formation viability (MTT-assay) 34

References 35

Experimental section

NMR Spectra

^{1}H NMR spectrum of **1a**

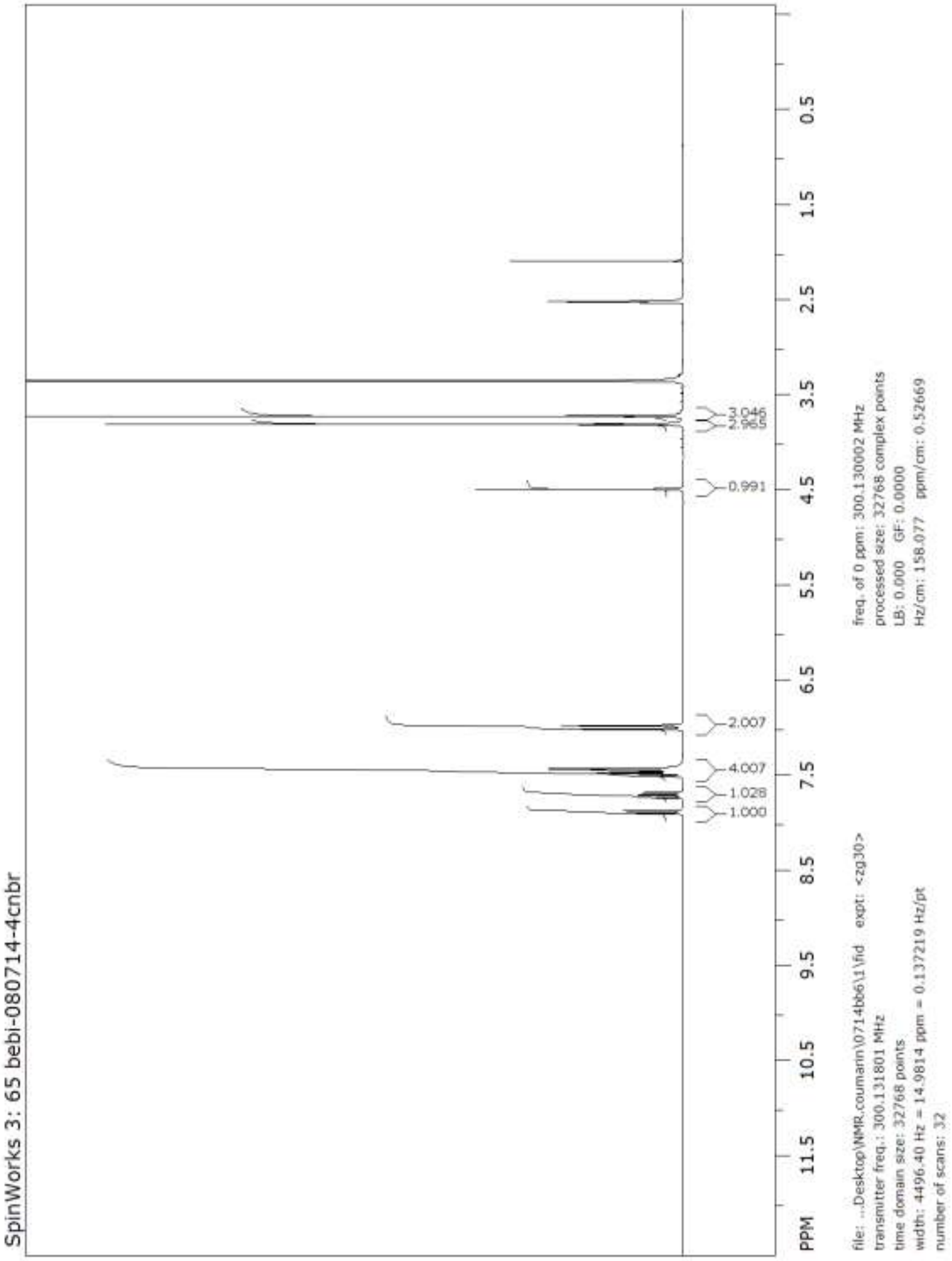

^{13}C NMR spectrum of **1a**

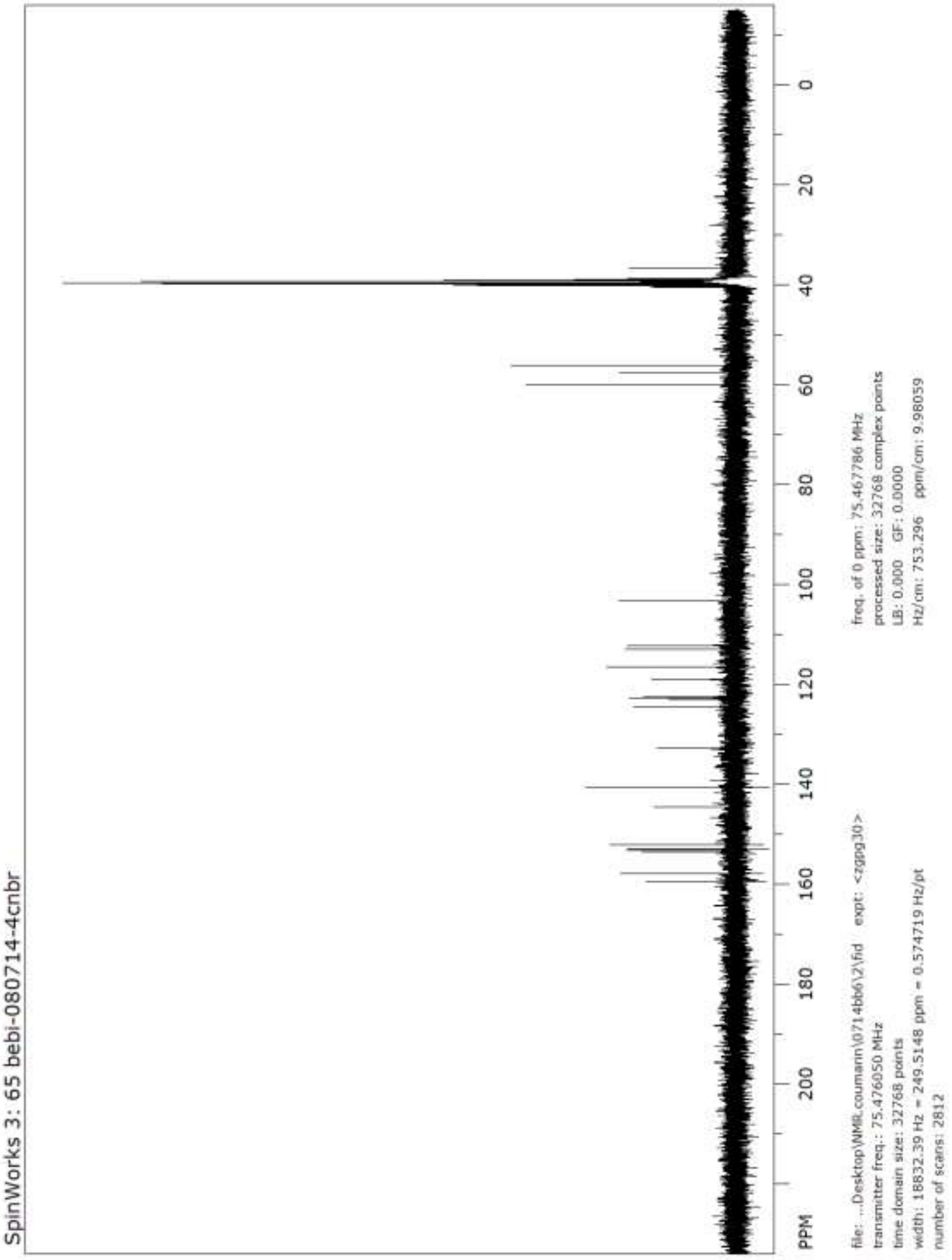

^{1}H NMR spectrum of **1b**

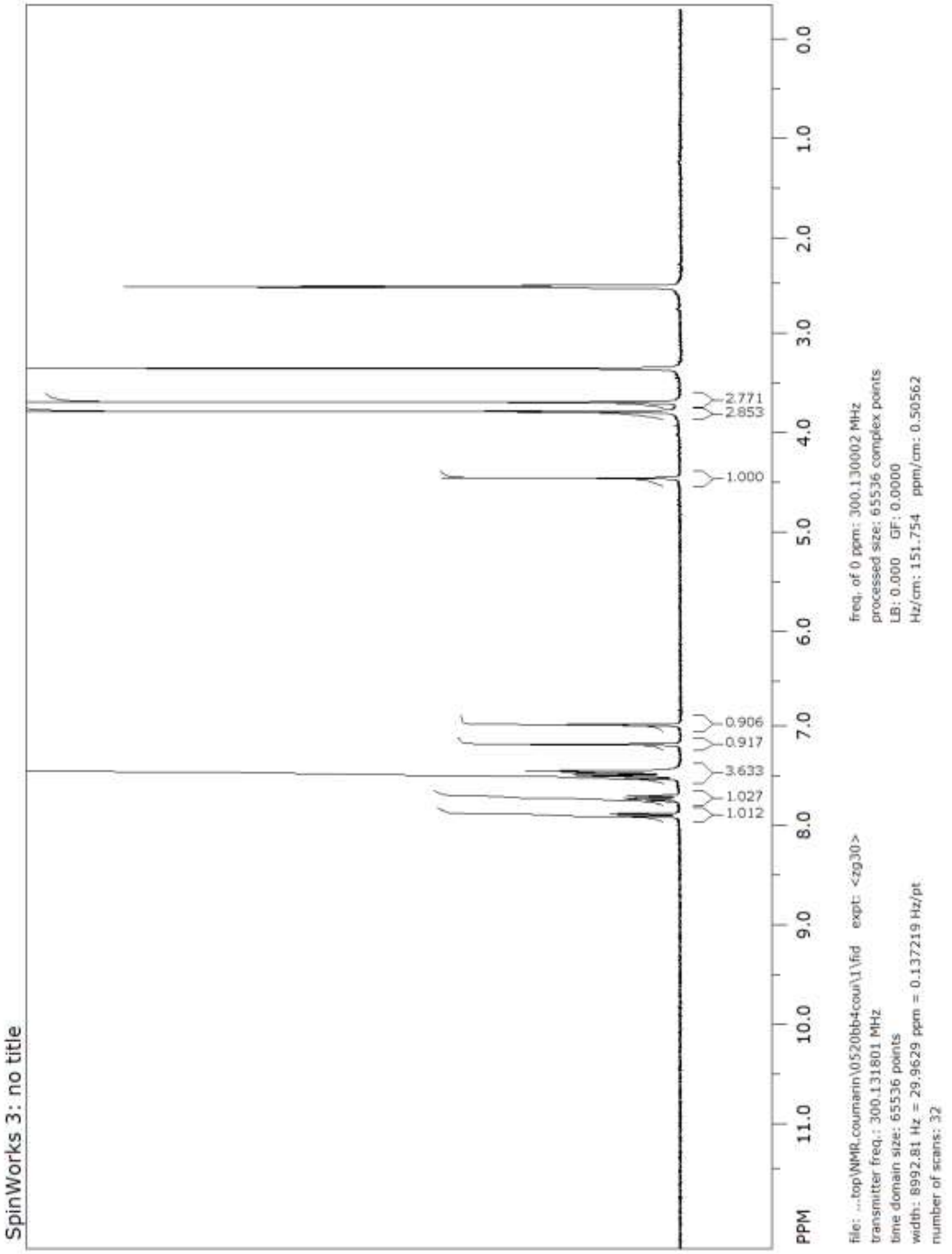

^{13}C NMR spectrum of **1b**

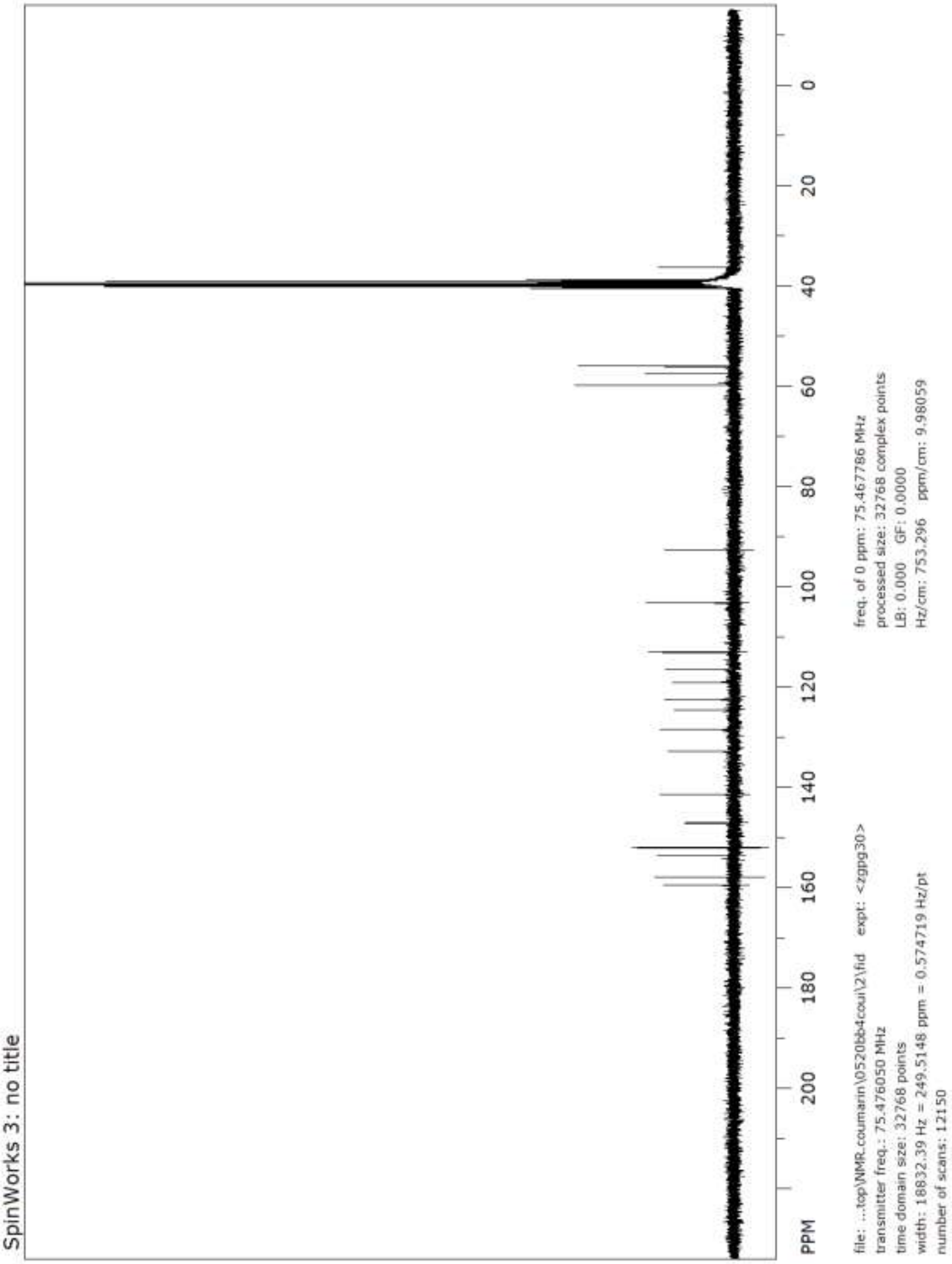

^{1}H NMR spectrum of **1c**

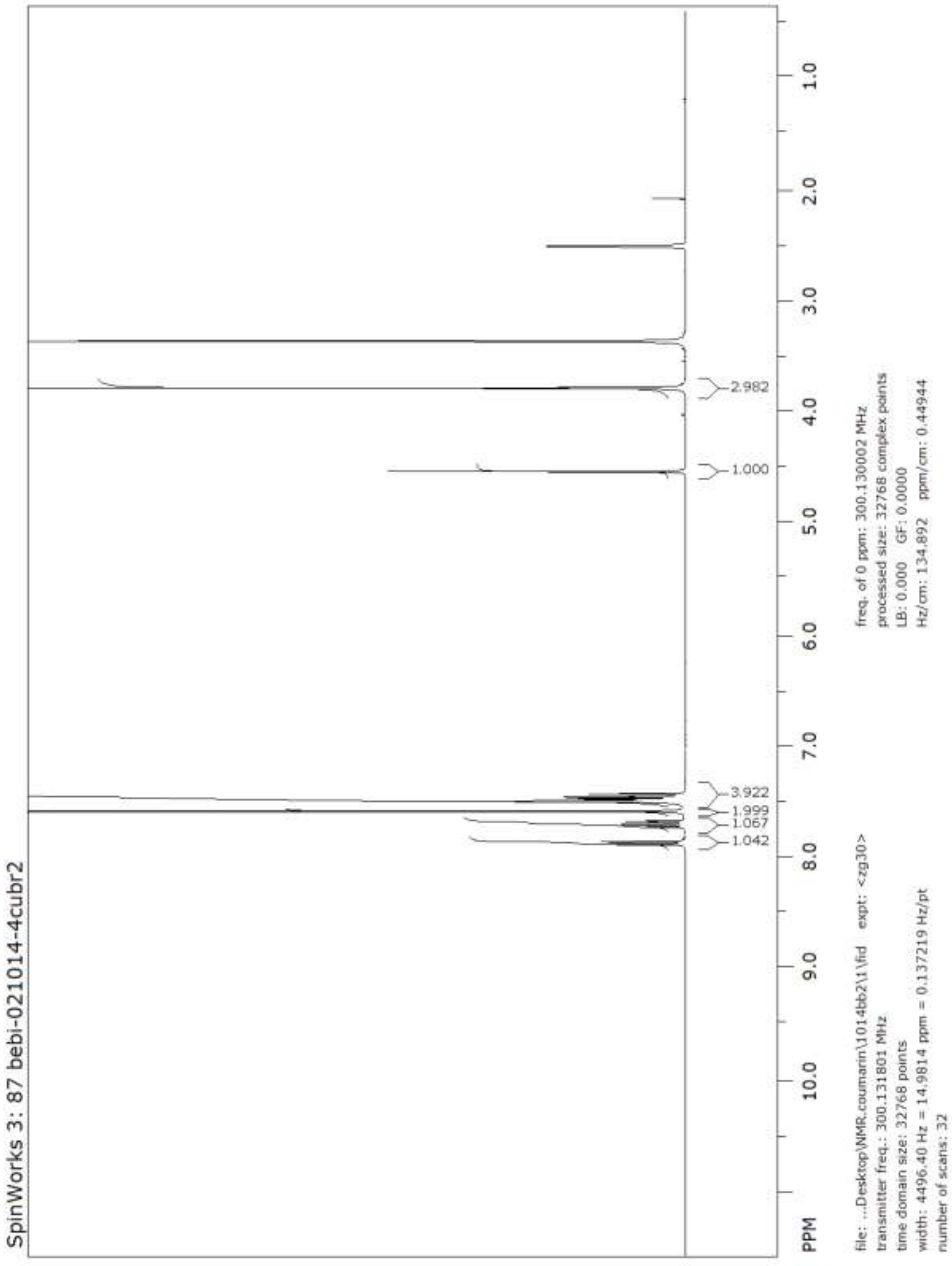

^{13}C NMR spectrum of **1c**

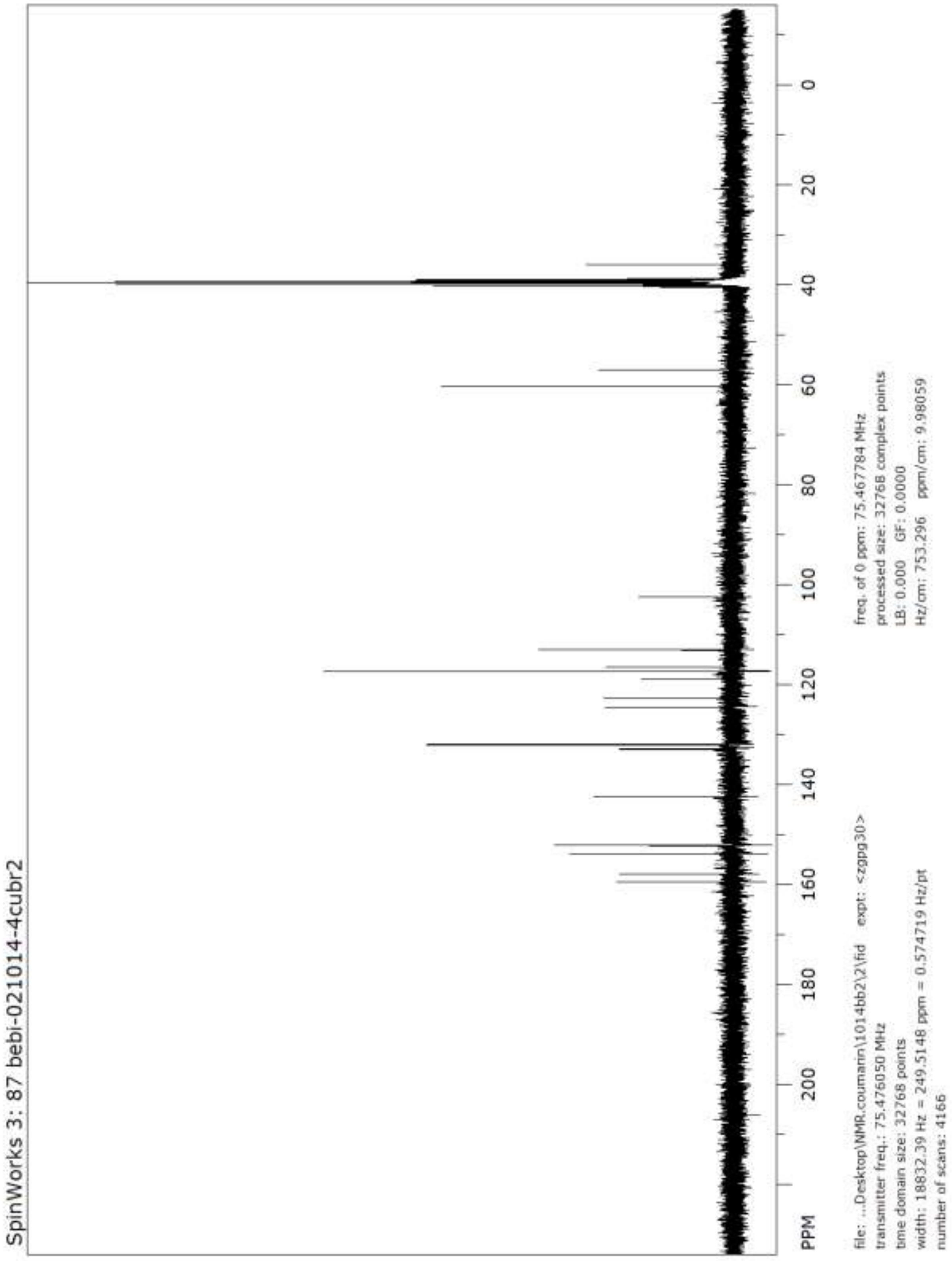

^{1}H NMR spectrum of **1d**

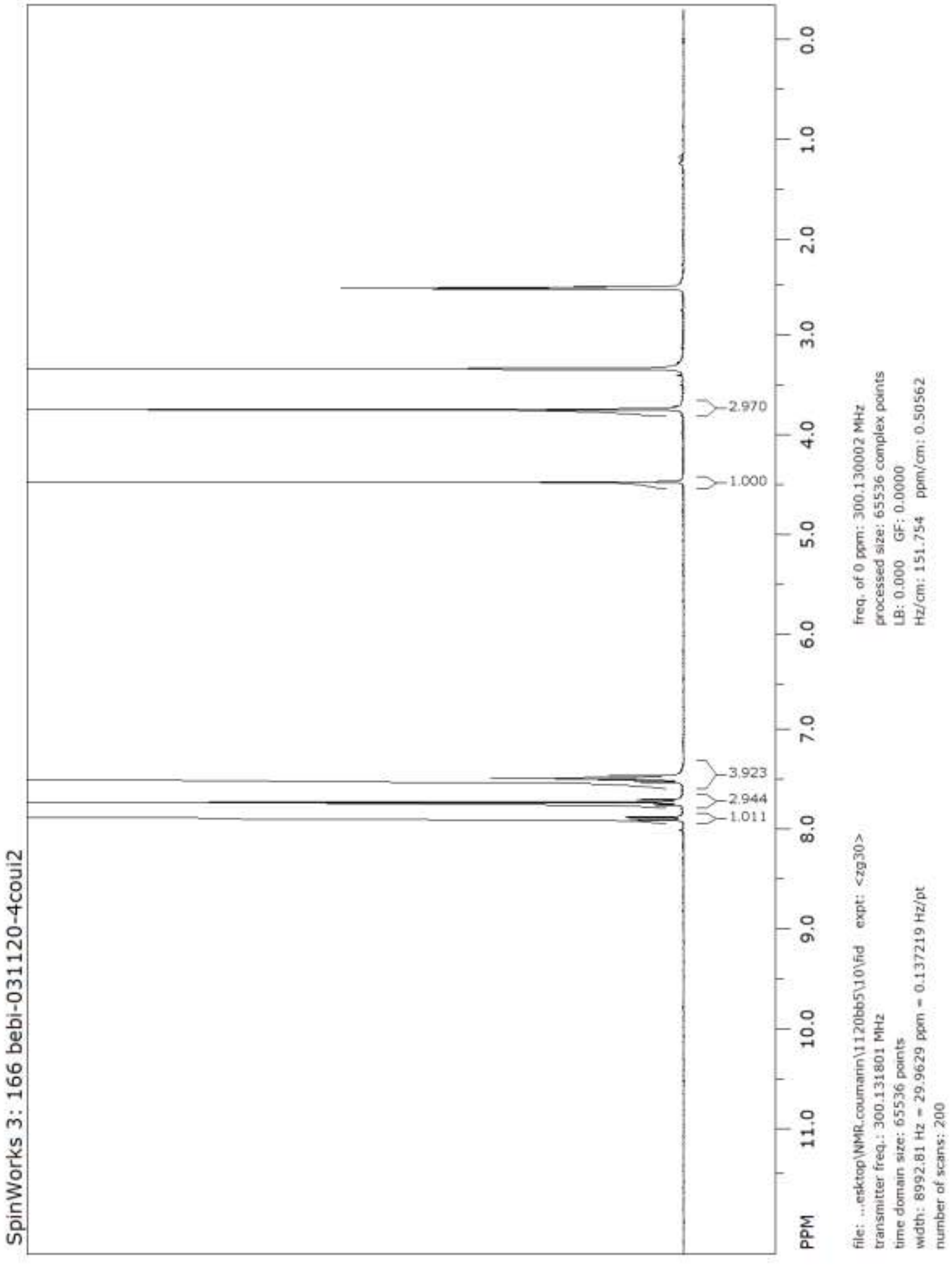

^{13}C NMR spectrum of **1d**

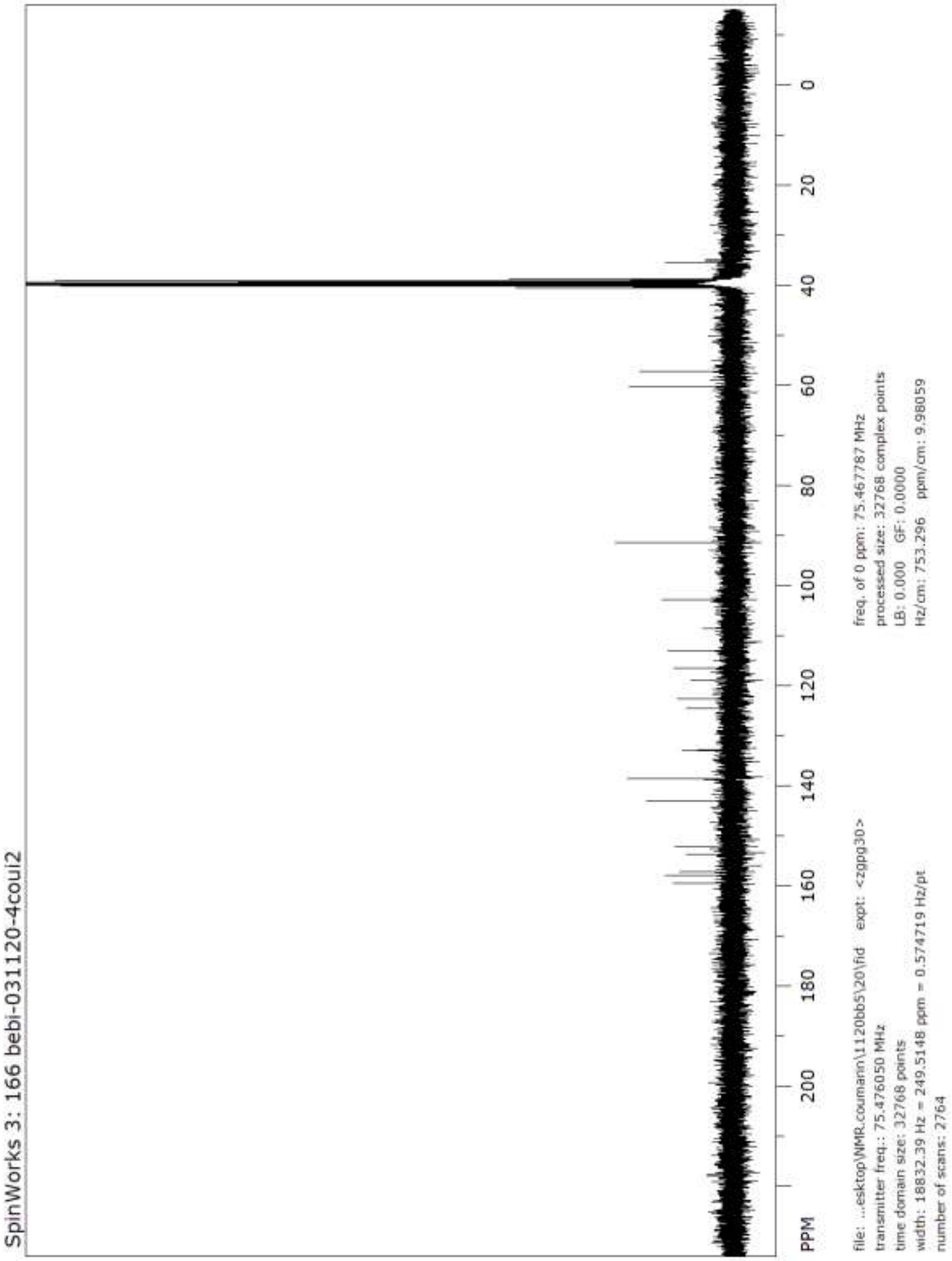

^{1}H NMR spectrum of **1e**

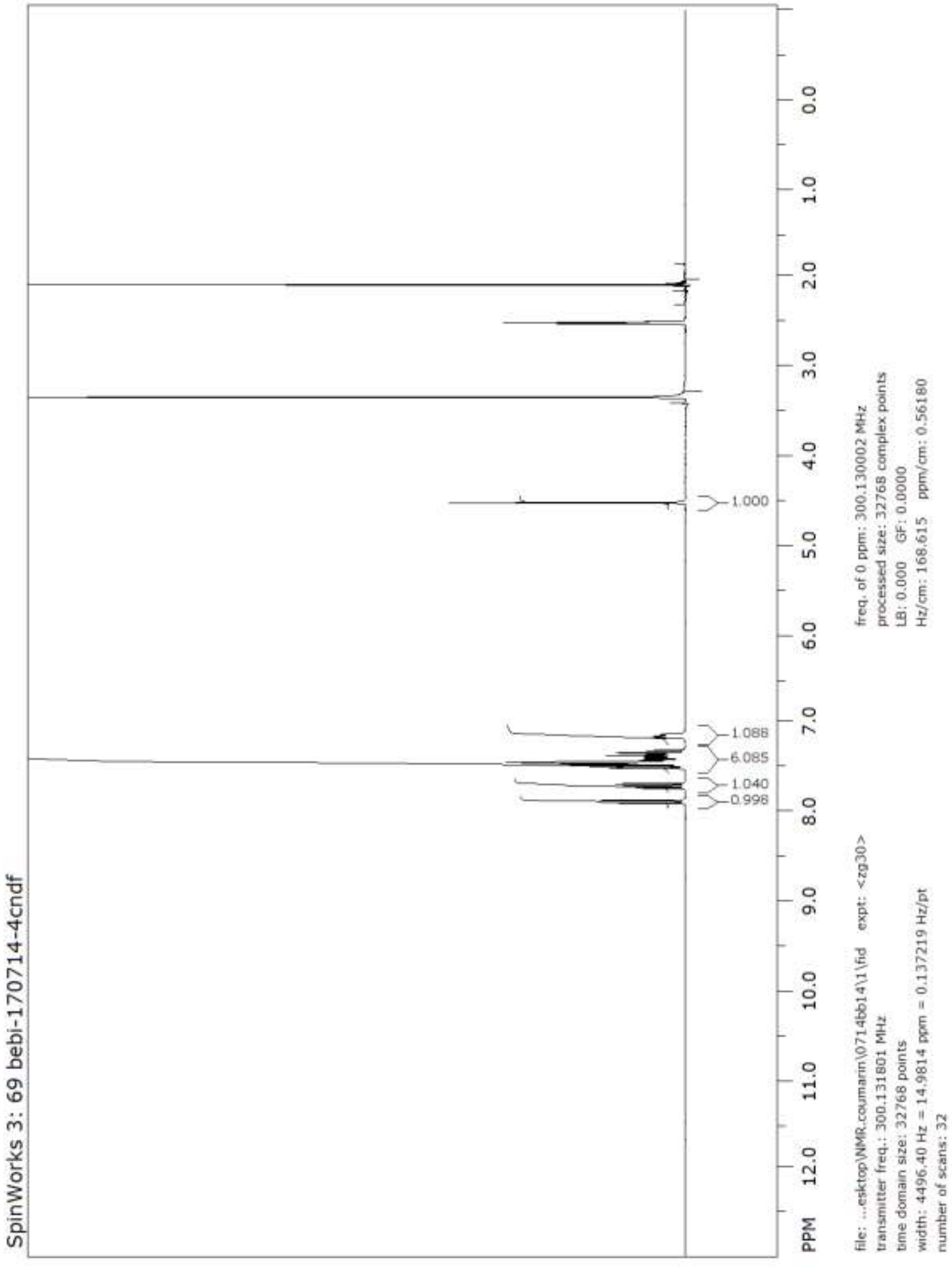

^{13}C NMR spectrum of **1e**

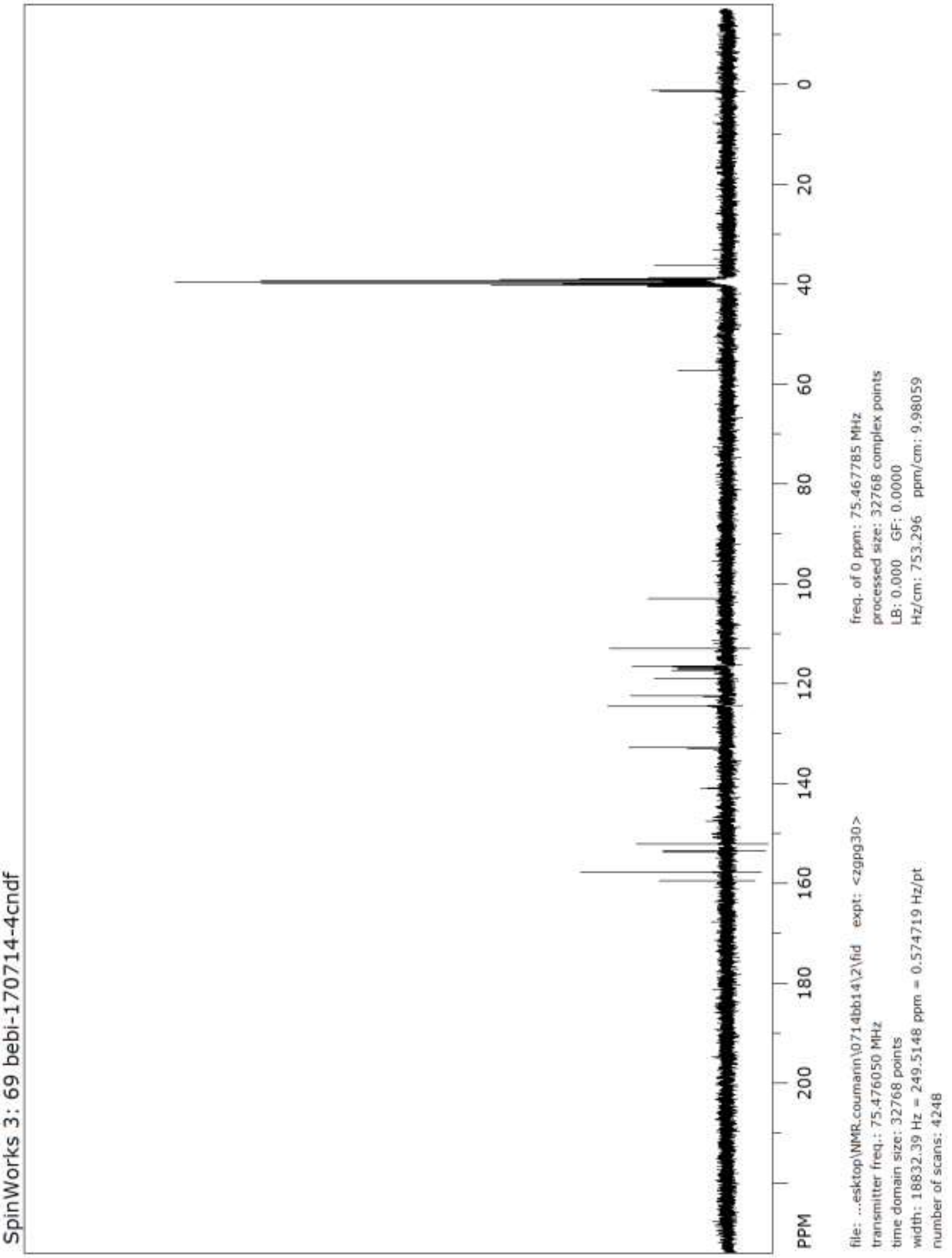

^{1}H NMR spectrum of **1f**

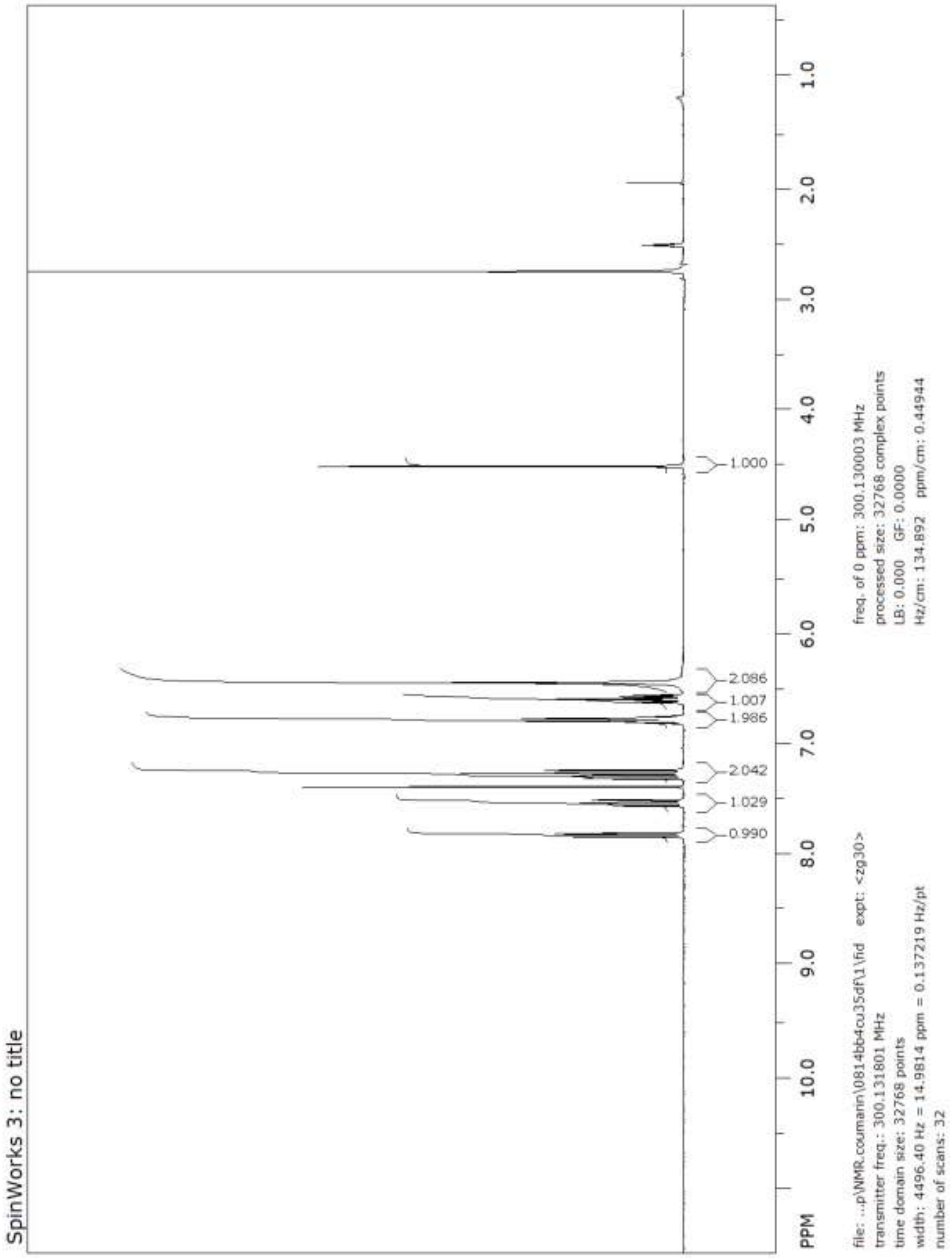

^{13}C NMR spectrum of **1f**

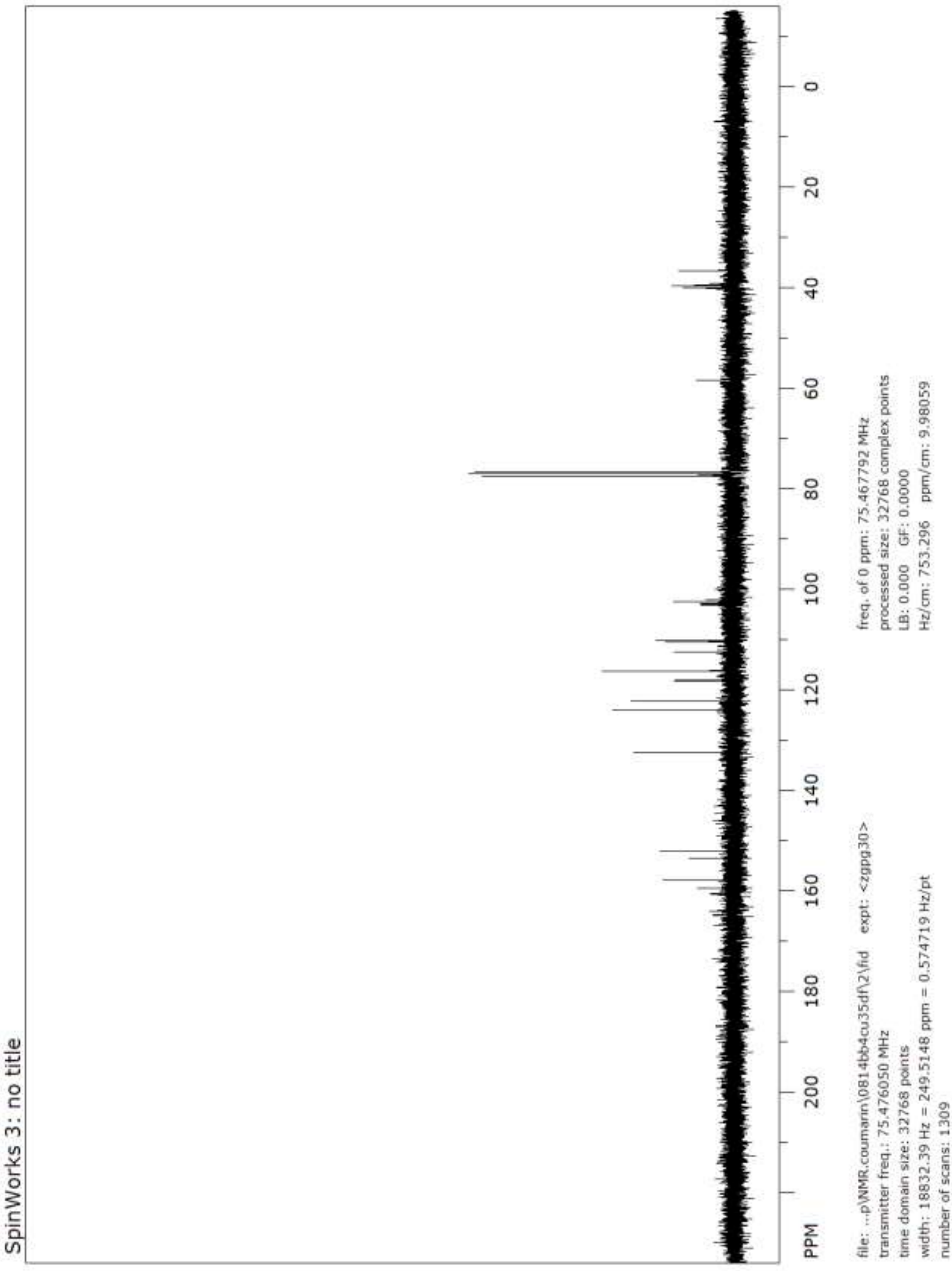

^{1}H NMR spectrum of **1g**

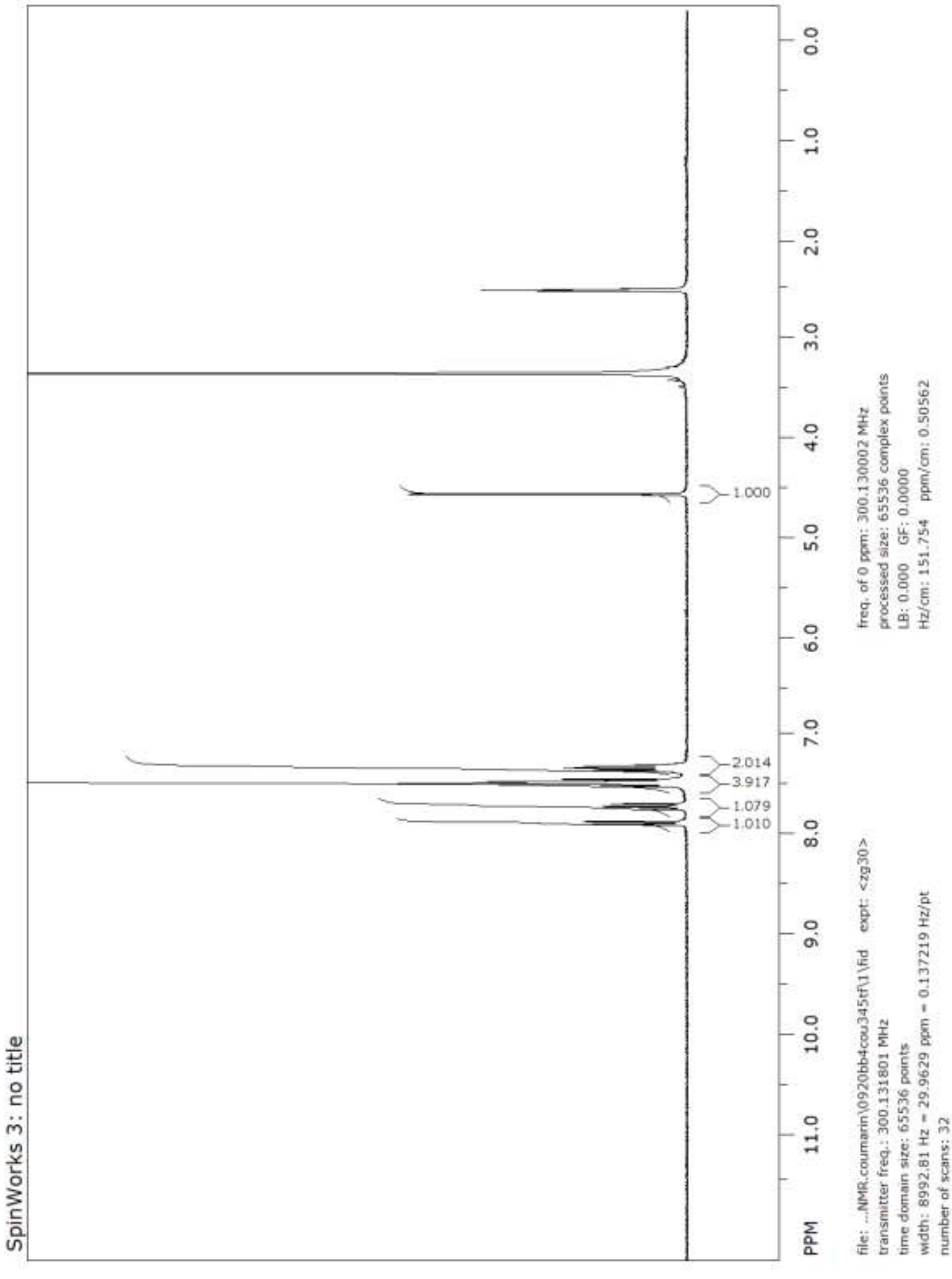

^{13}C NMR spectrum of **1g**

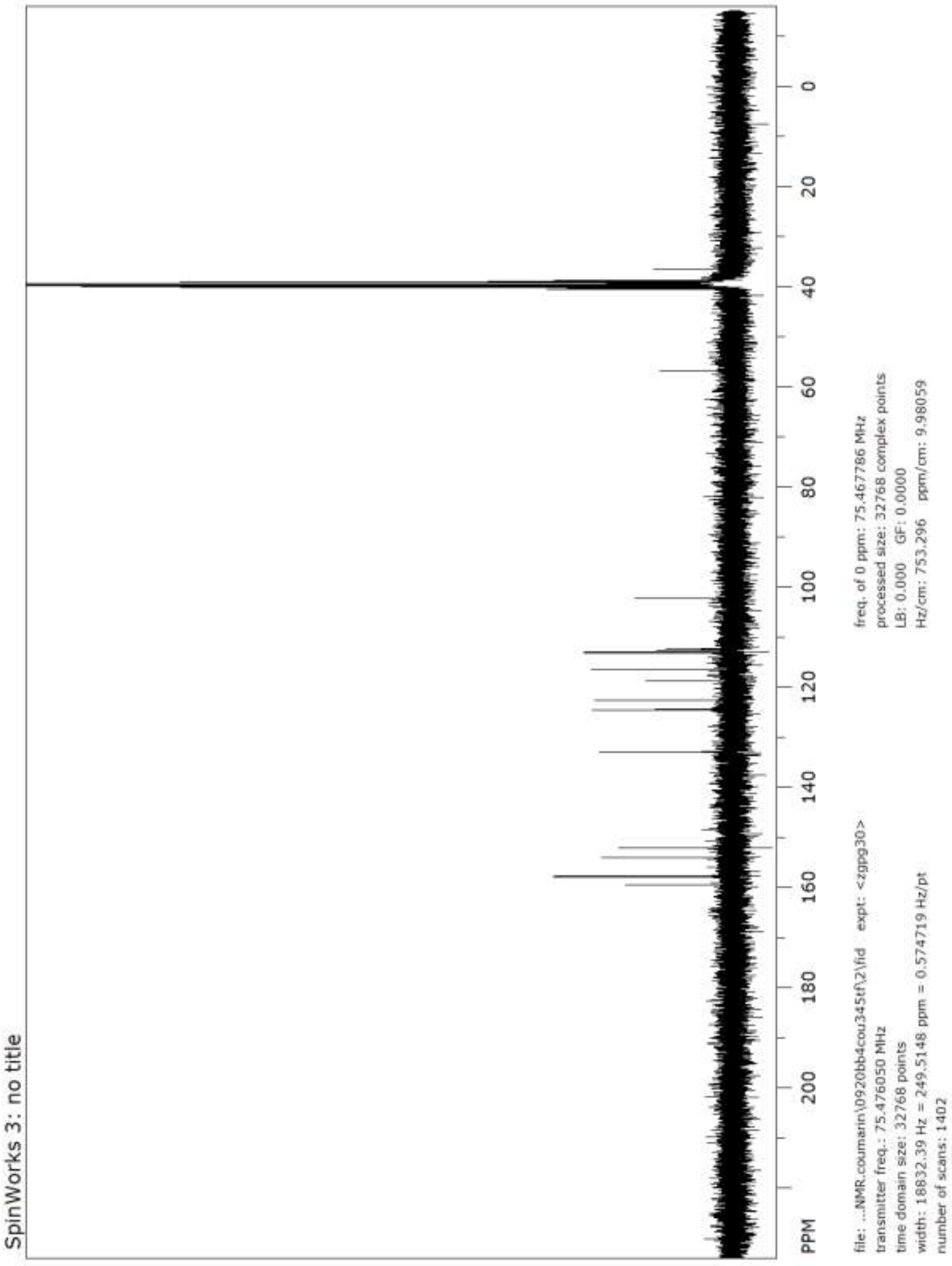

^{1}H NMR spectrum of **1h**

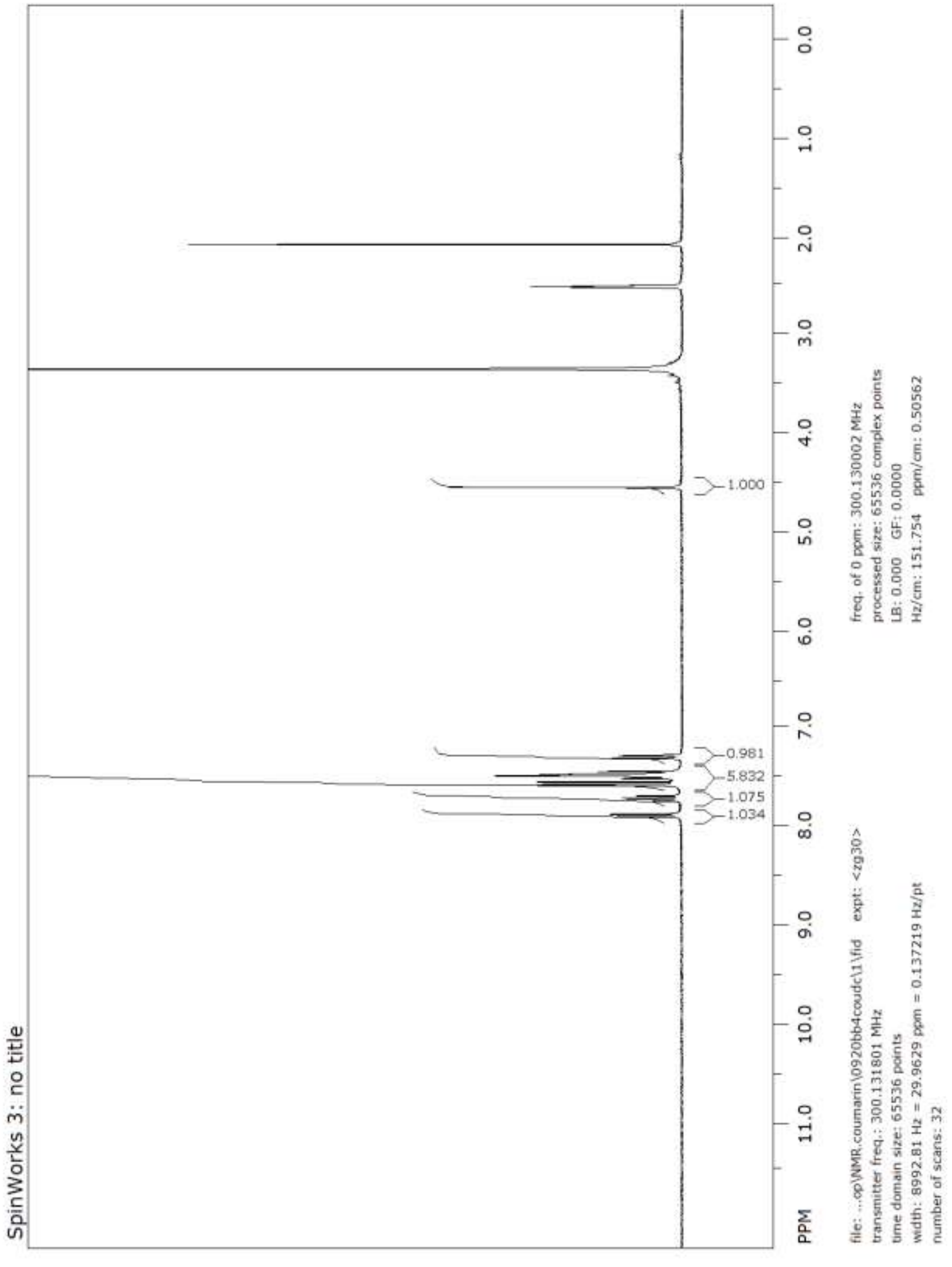

^{13}C NMR spectrum of **1h**

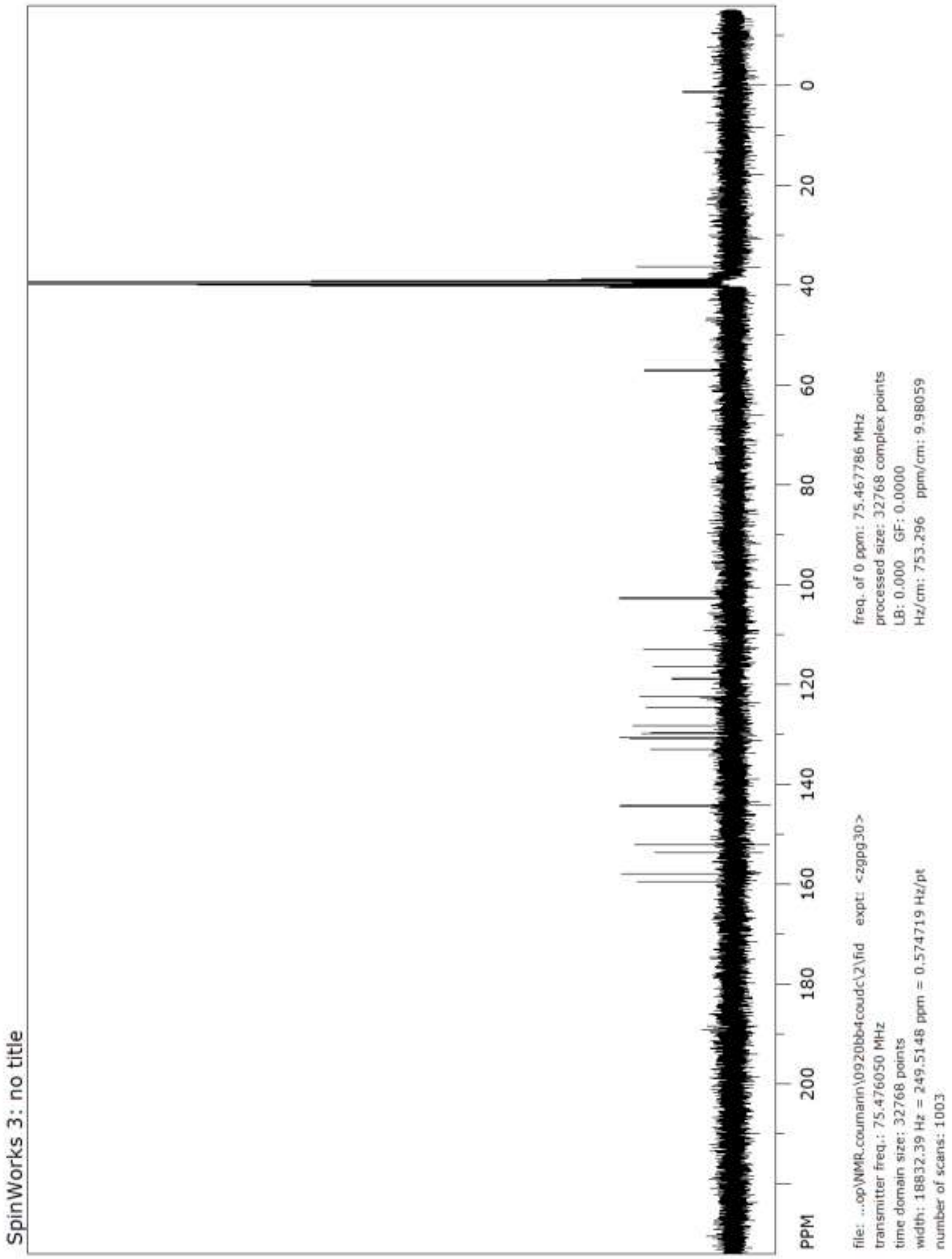

^{1}H NMR spectrum of **1i**

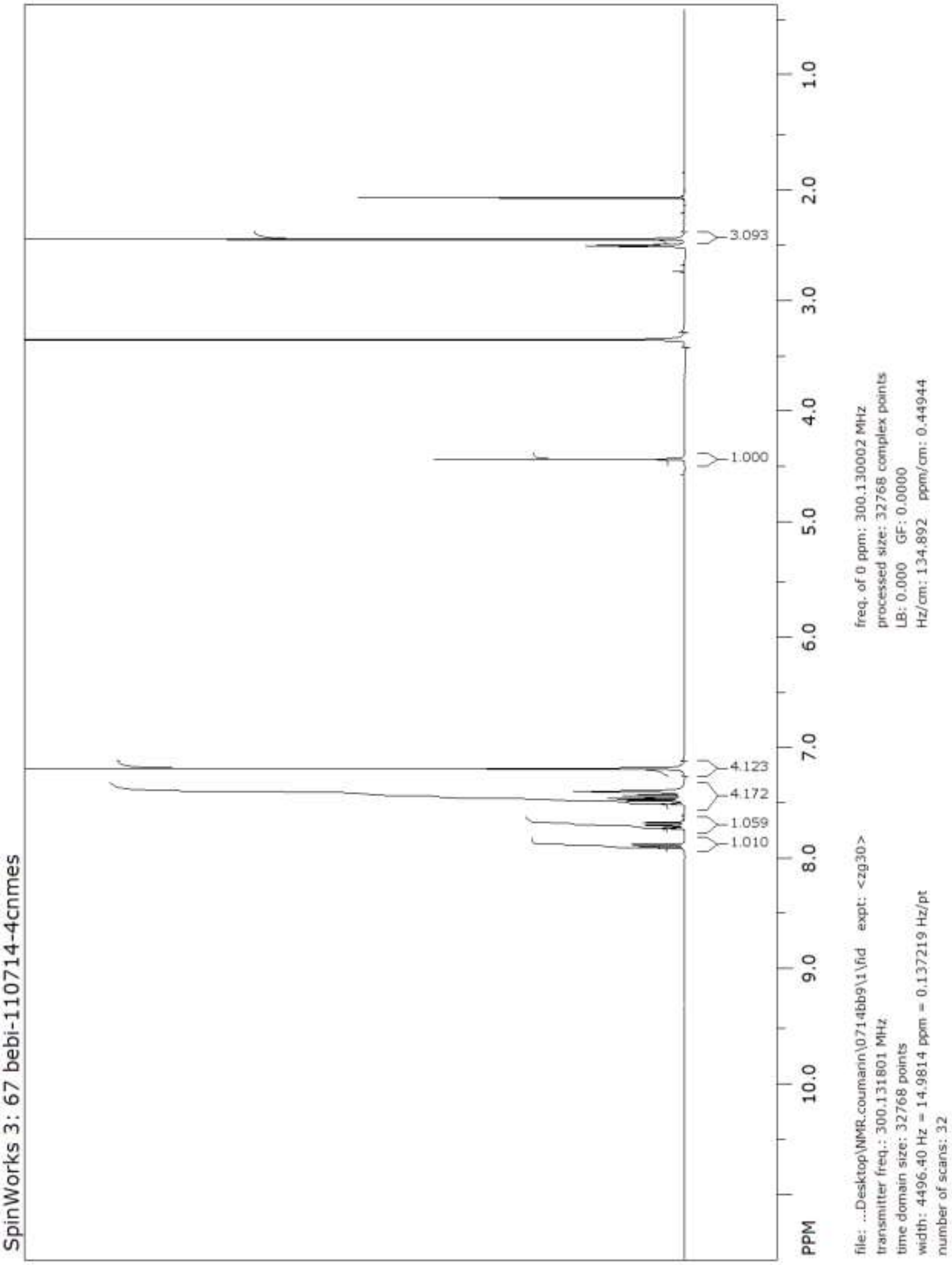

^{13}C NMR spectrum of **1i**

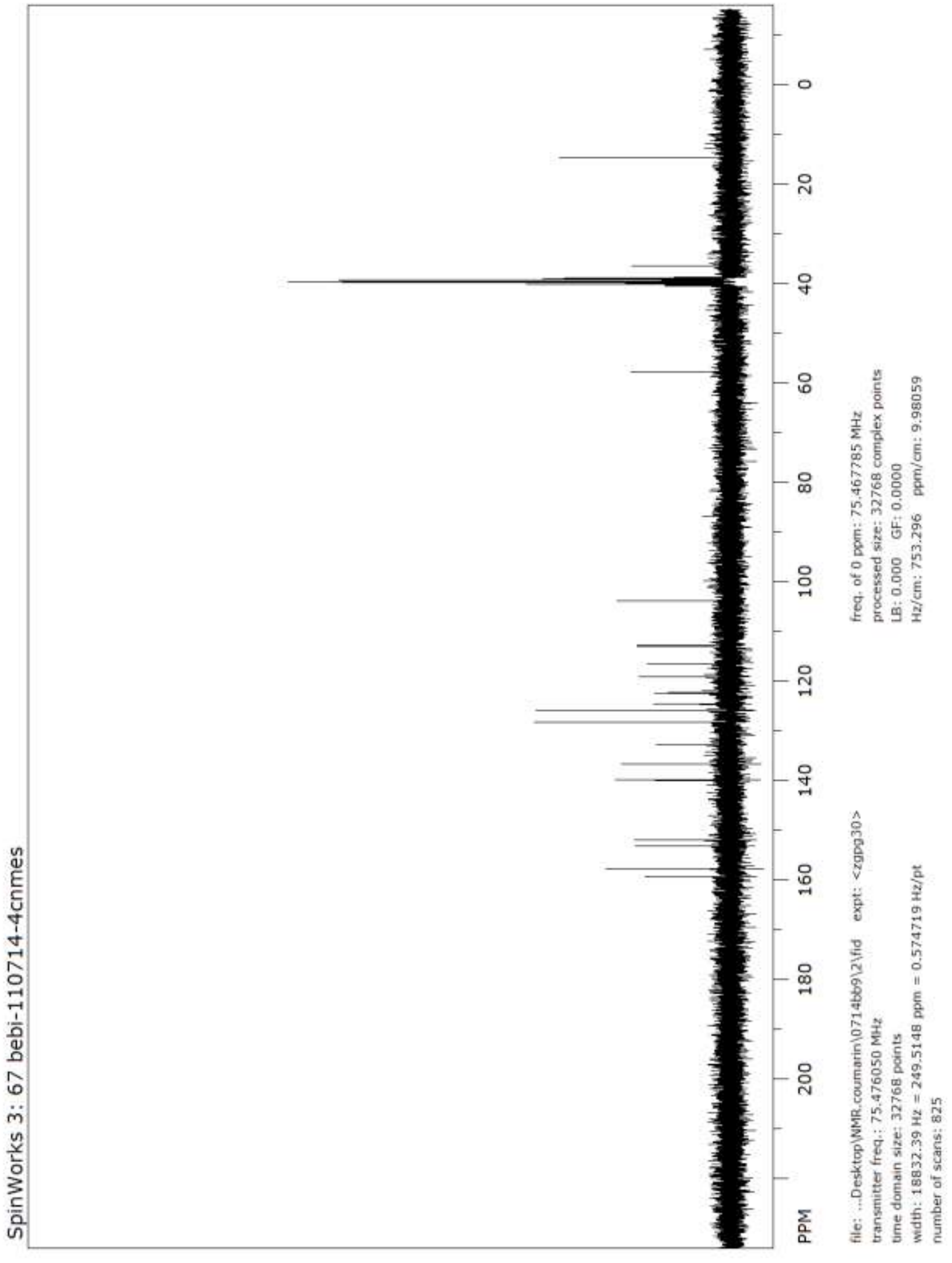

^{1}H NMR spectrum of **1j**

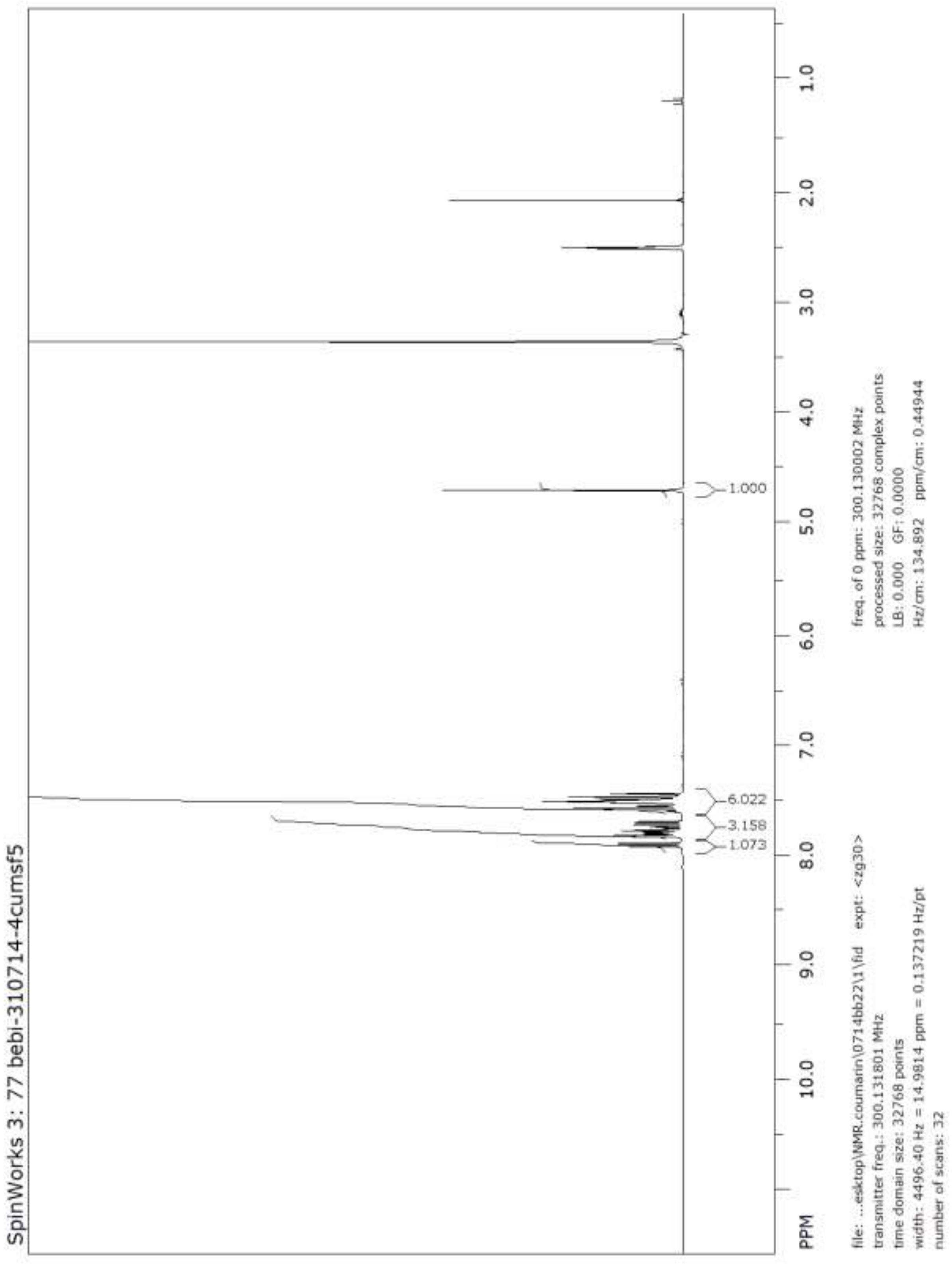

^{13}C NMR spectrum of **1j**

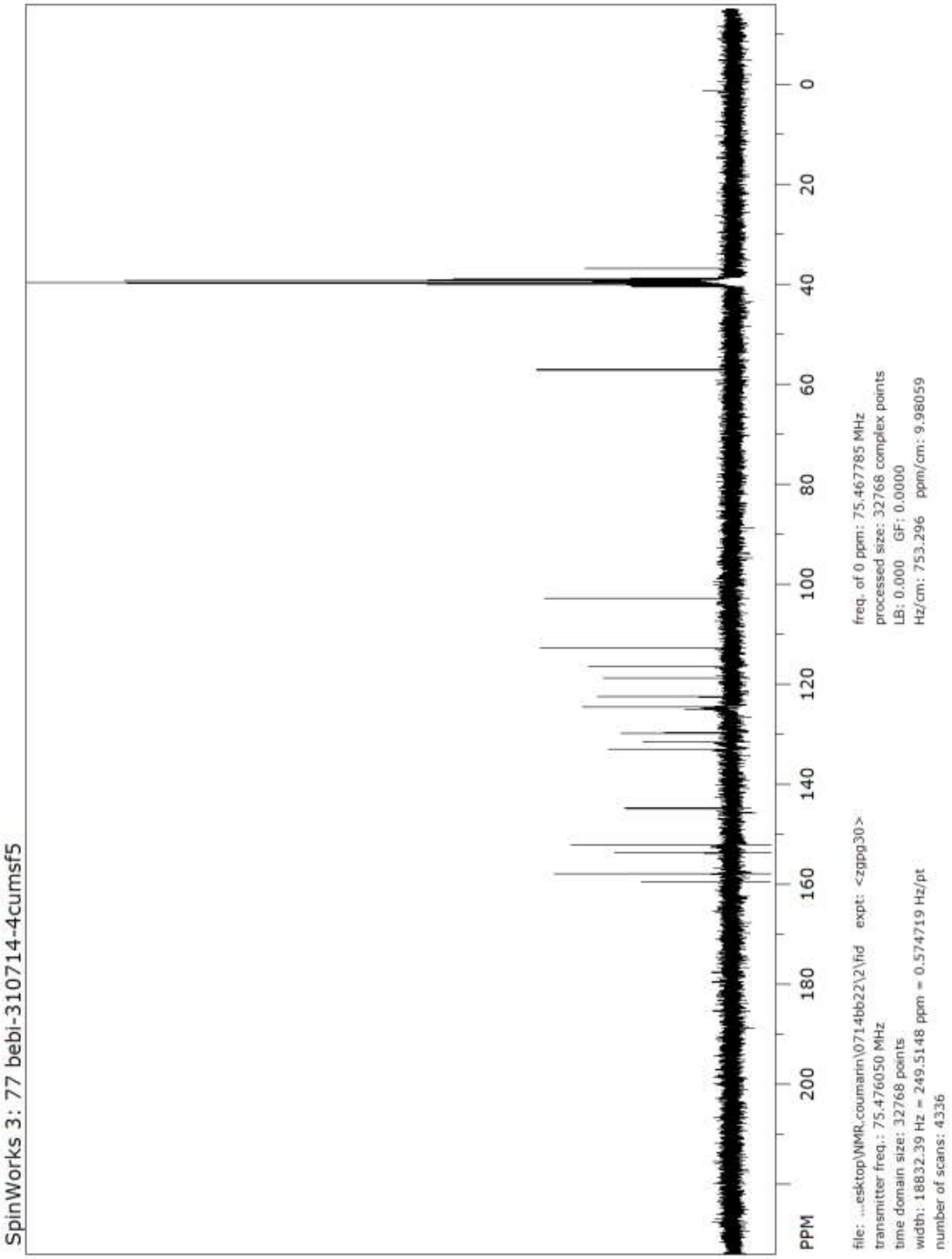

^{1}H NMR spectrum of **1k**

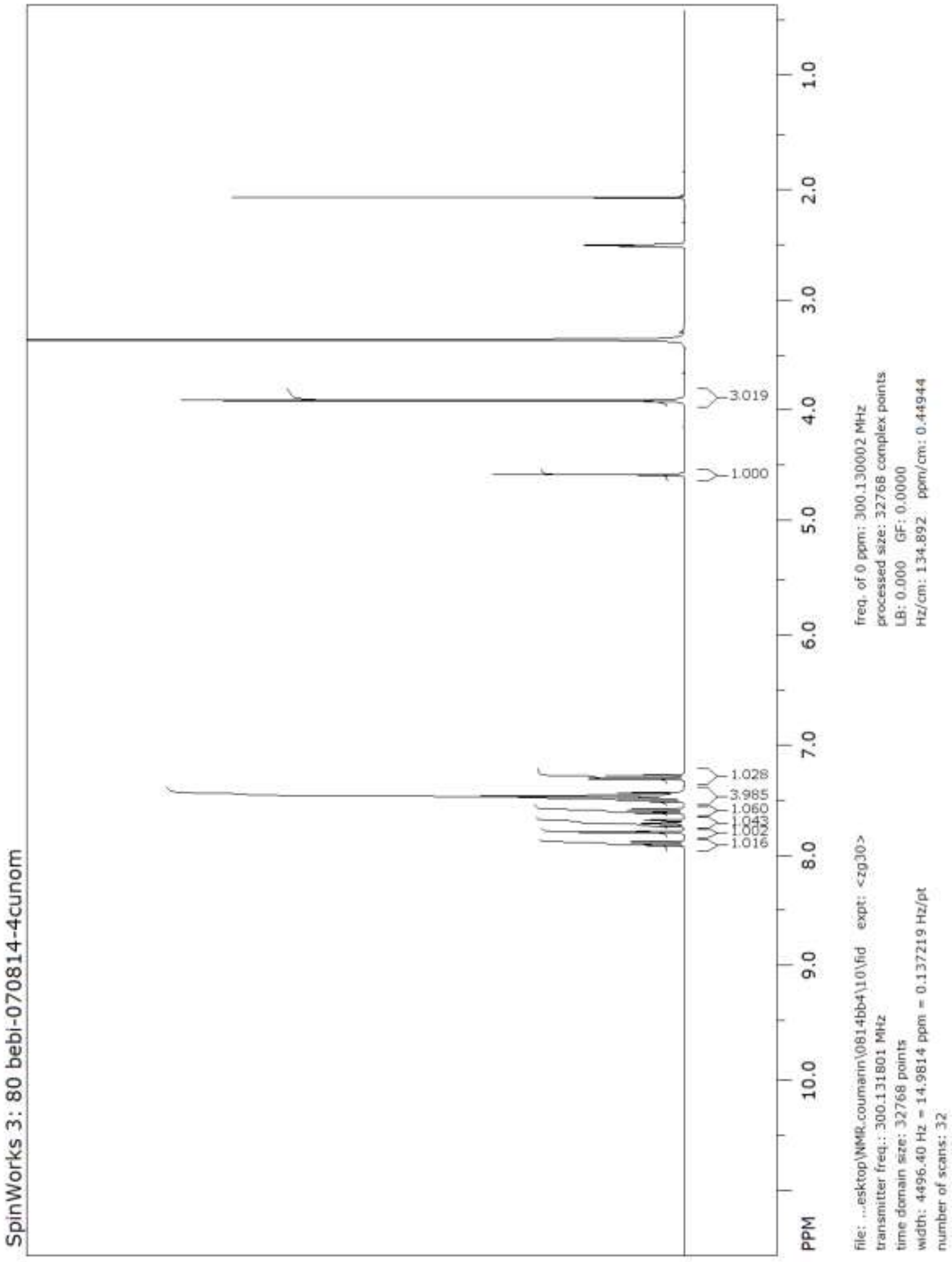

^{13}C NMR spectrum of **1k**

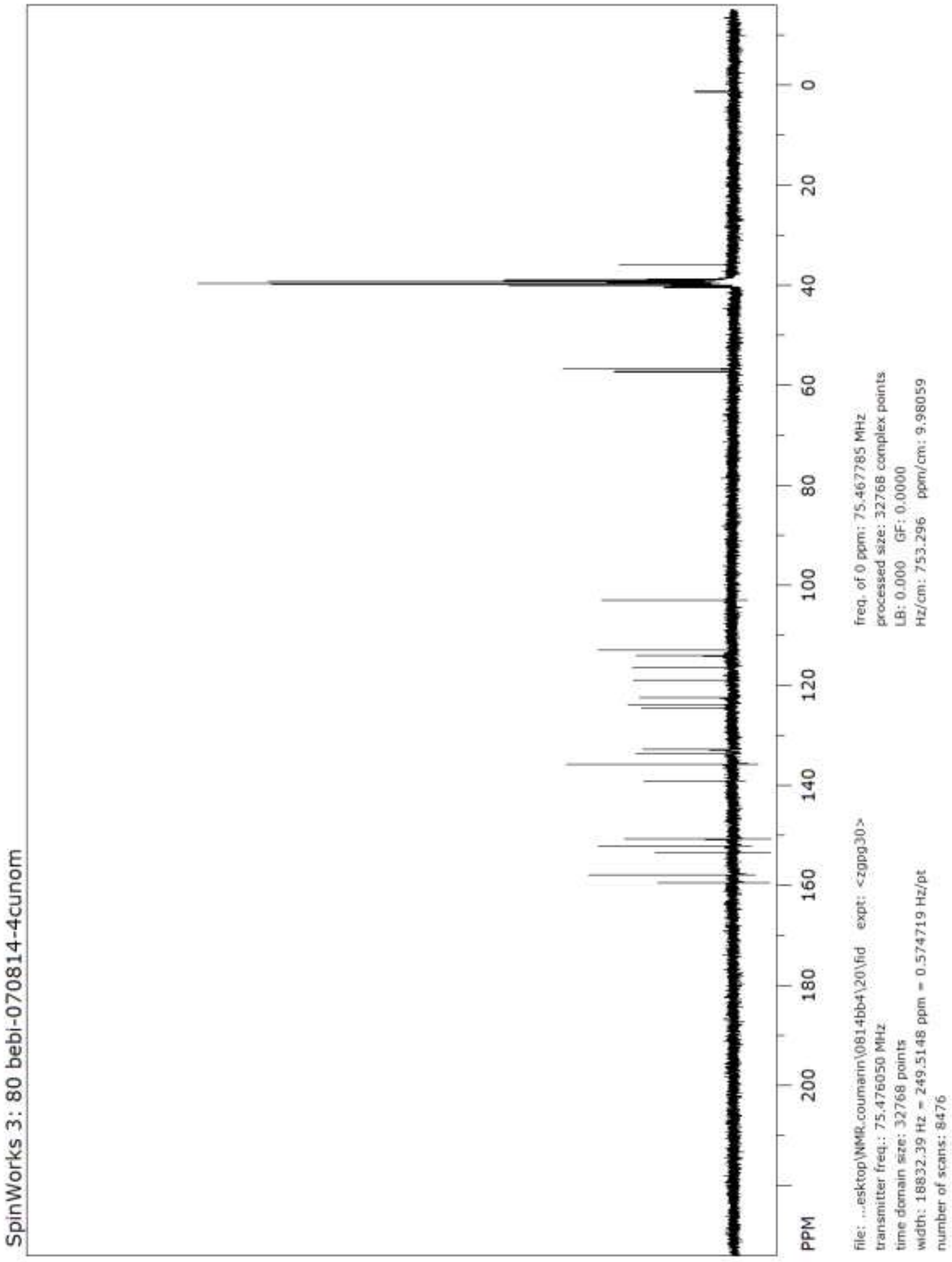

^{1}H NMR spectrum of **1l**

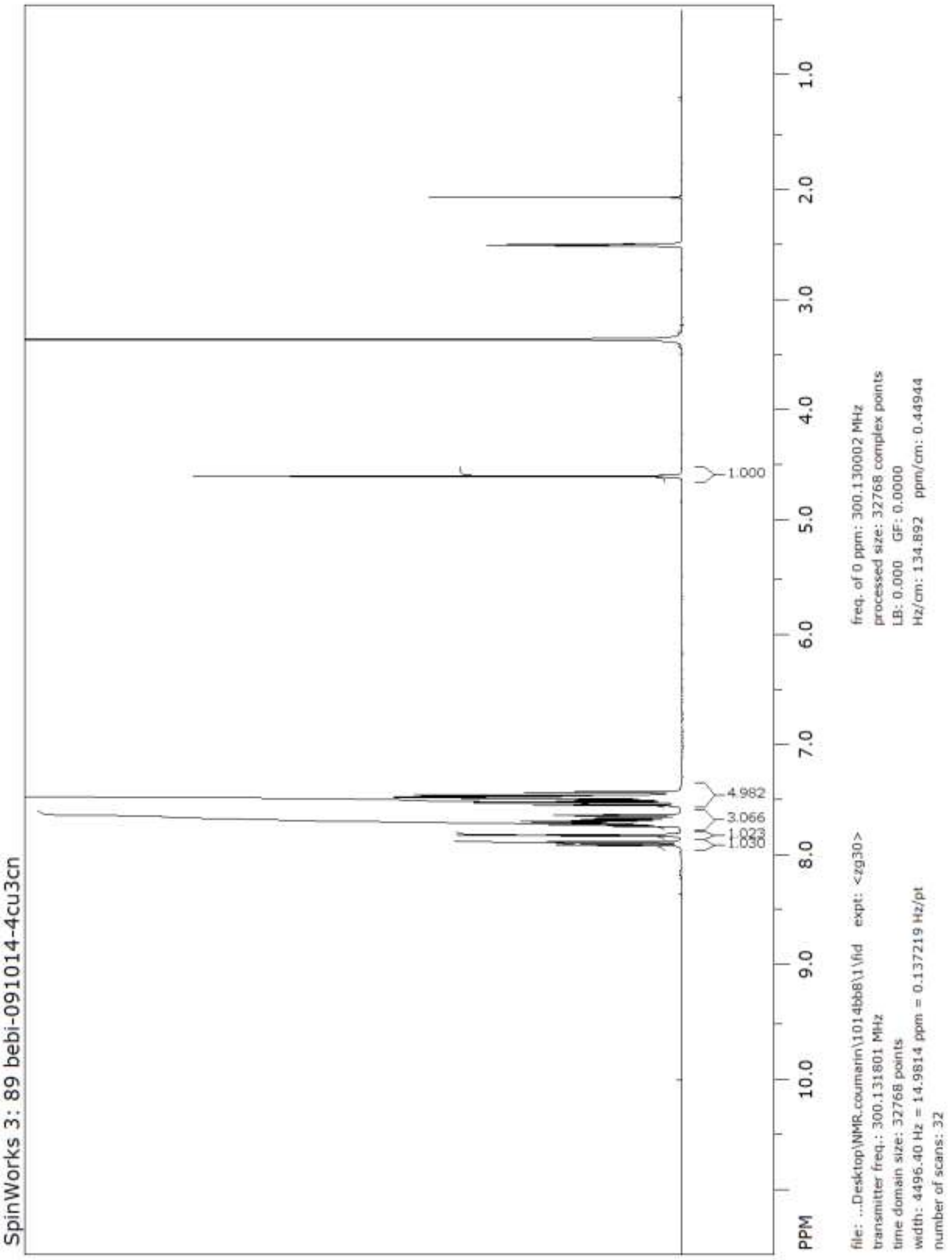

^{13}C NMR spectrum of **1l**

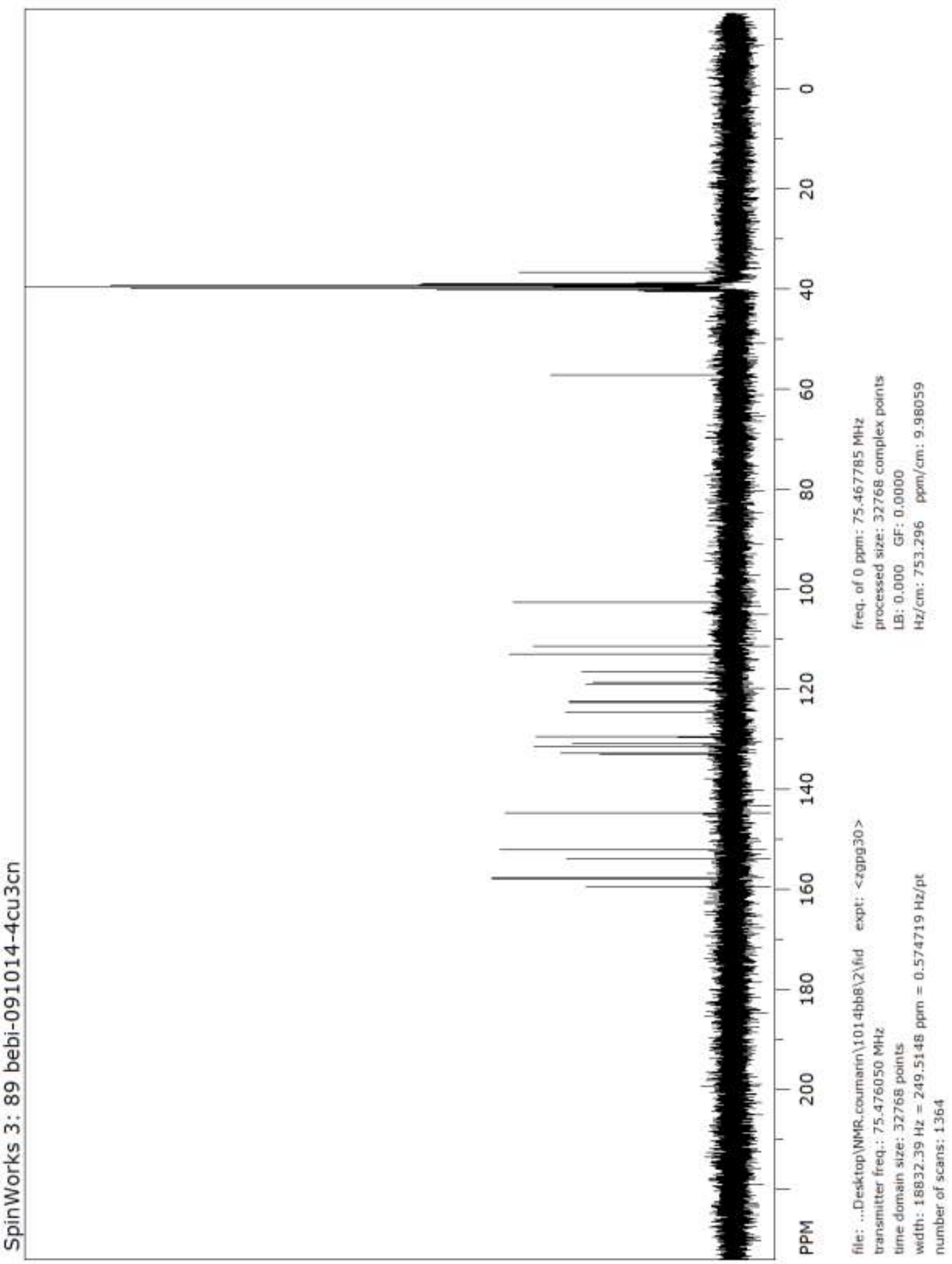

^{1}H NMR spectrum of **1m**

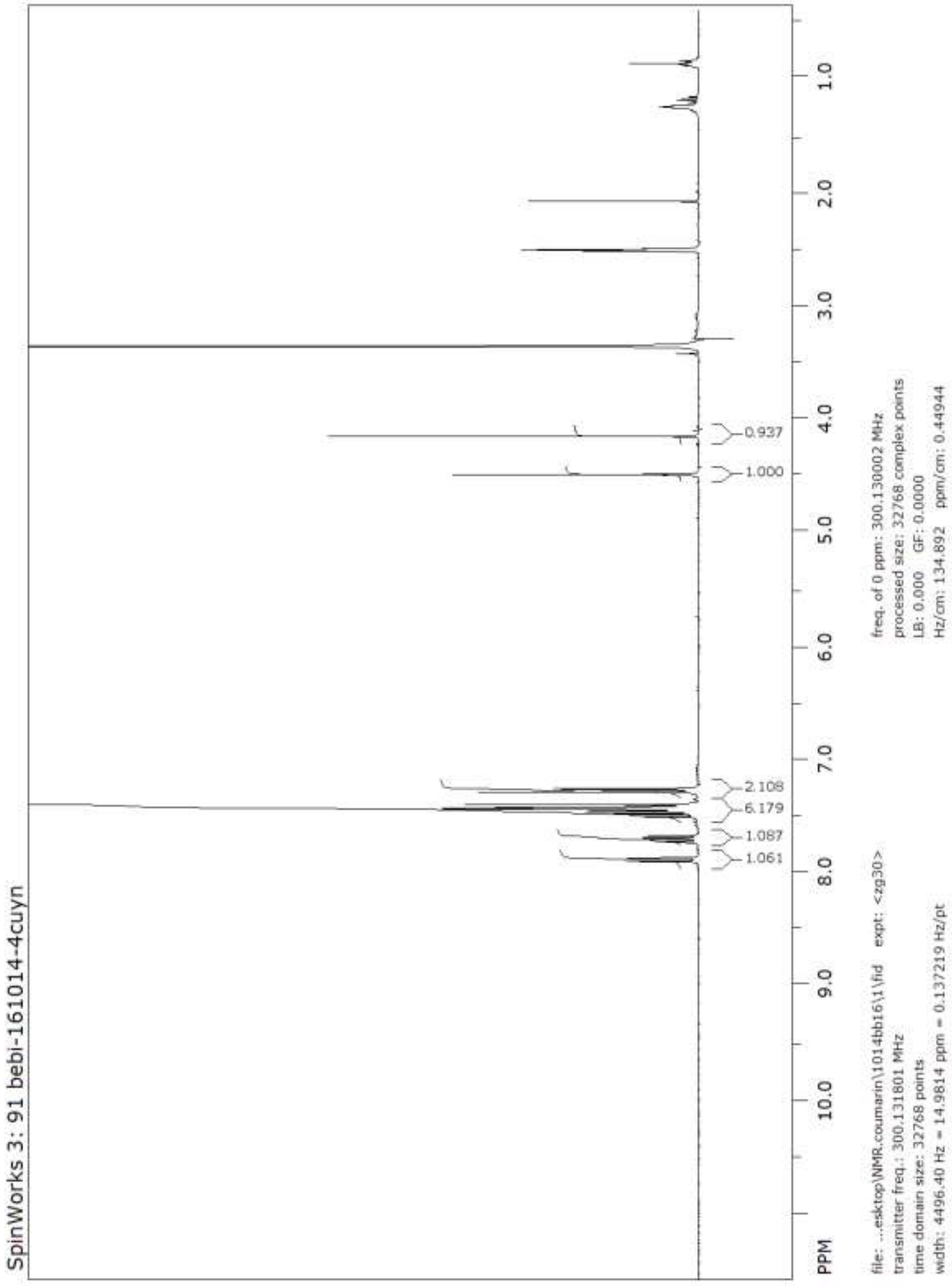

^{13}C NMR spectrum of **1m**

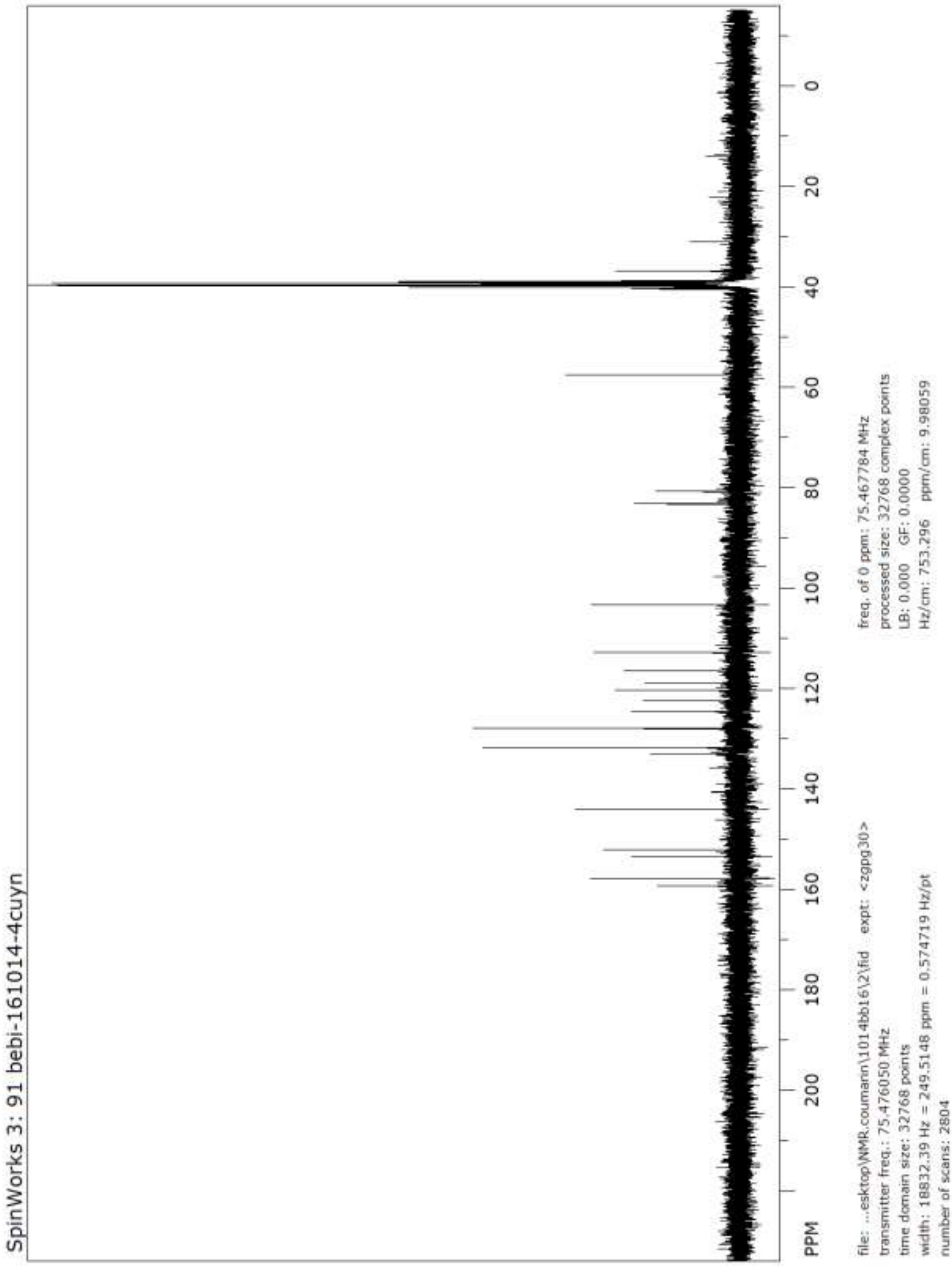

^{1}H NMR spectrum of **1n**

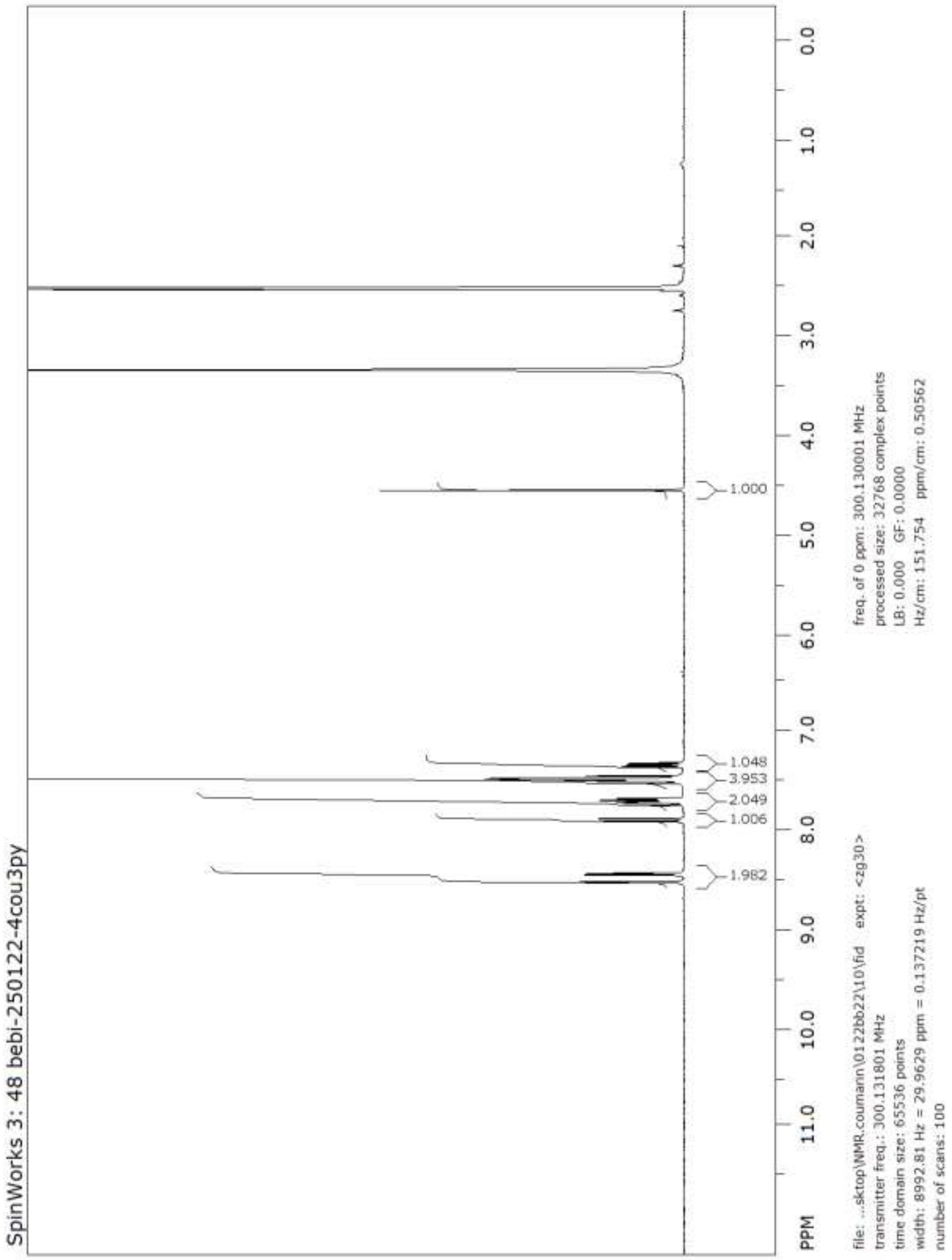

^{1}H NMR spectrum of **1m**

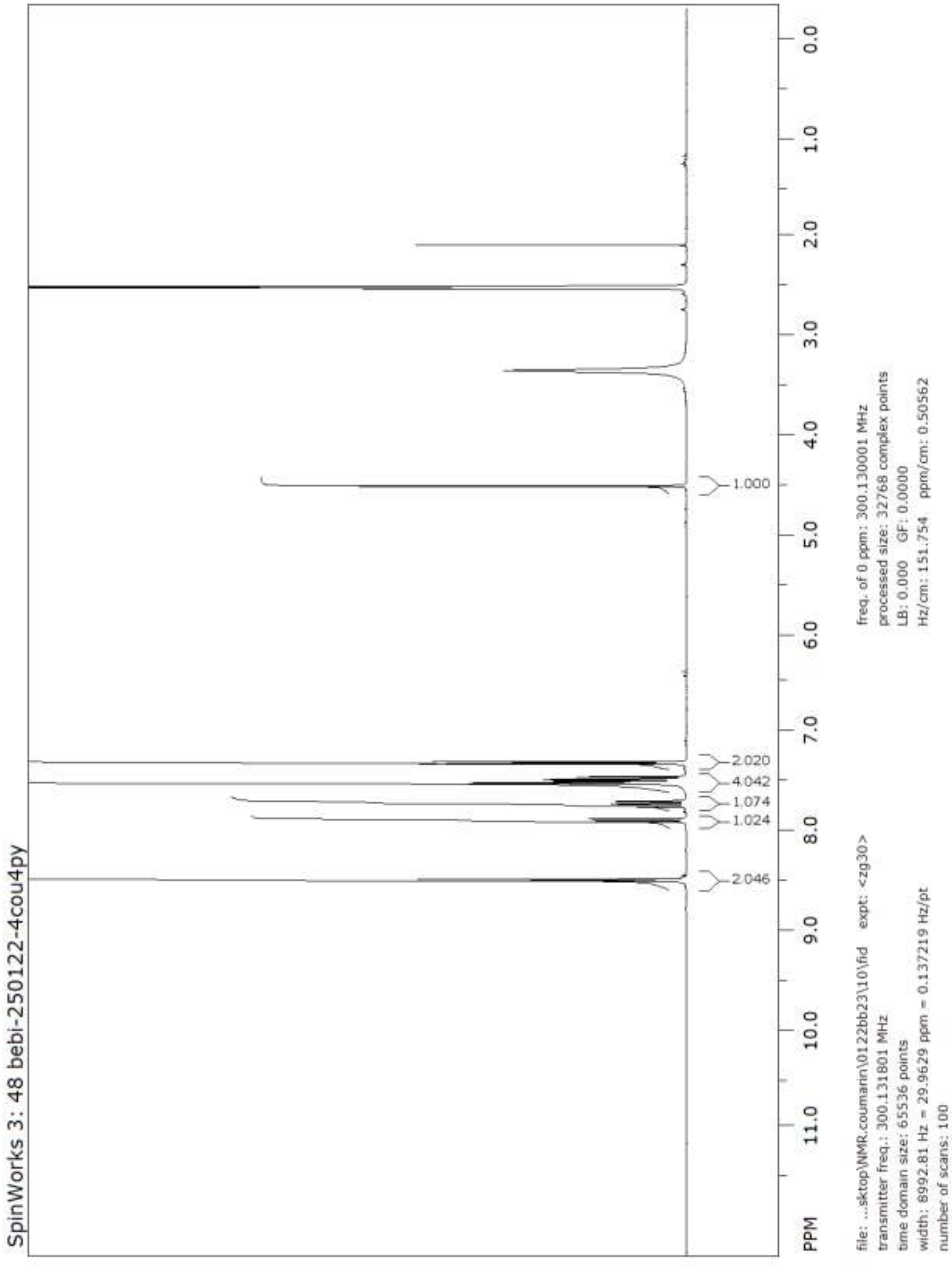

^{1}H NMR spectrum of **1o**

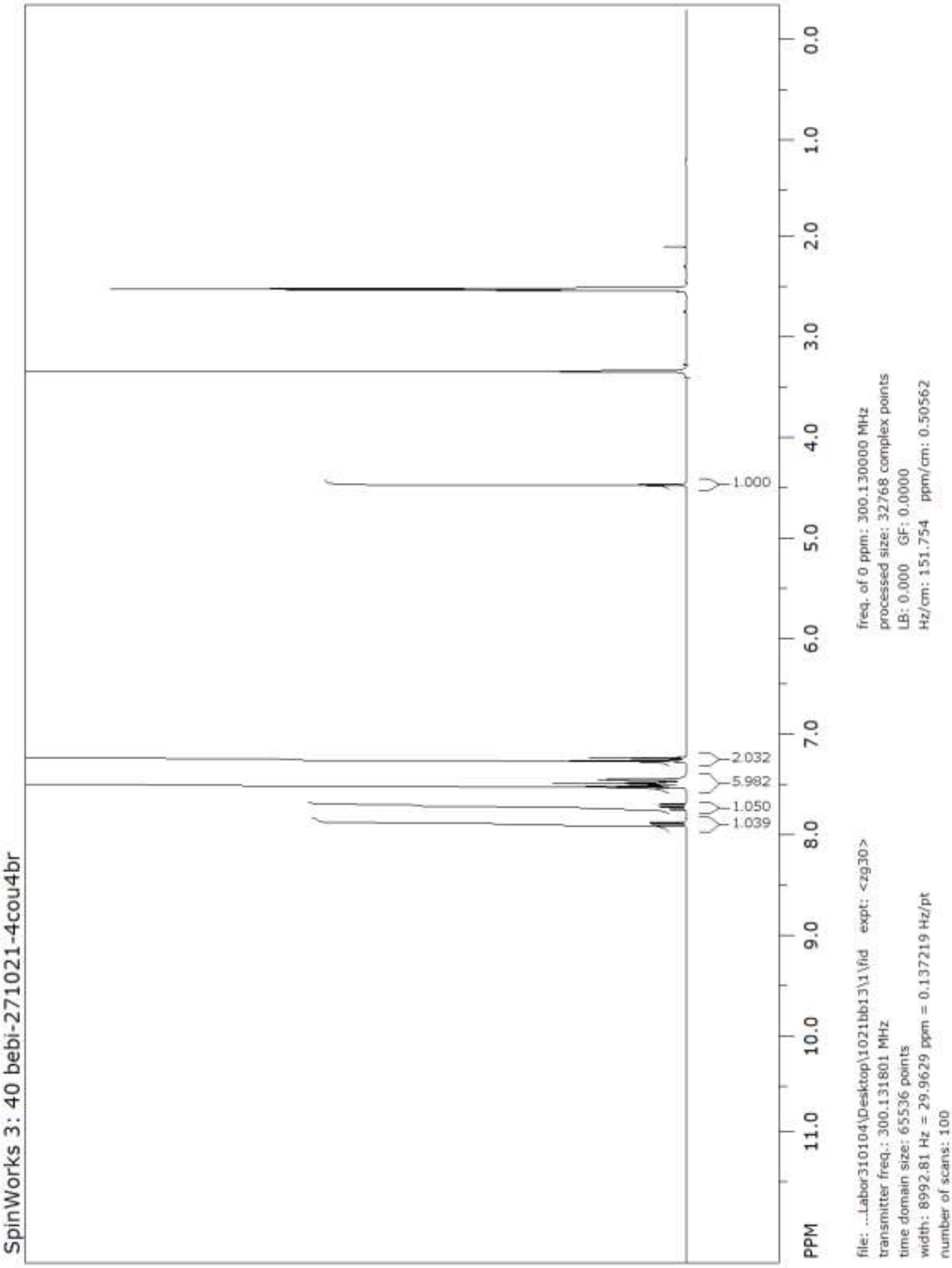

^{1}H NMR spectrum of **1p**

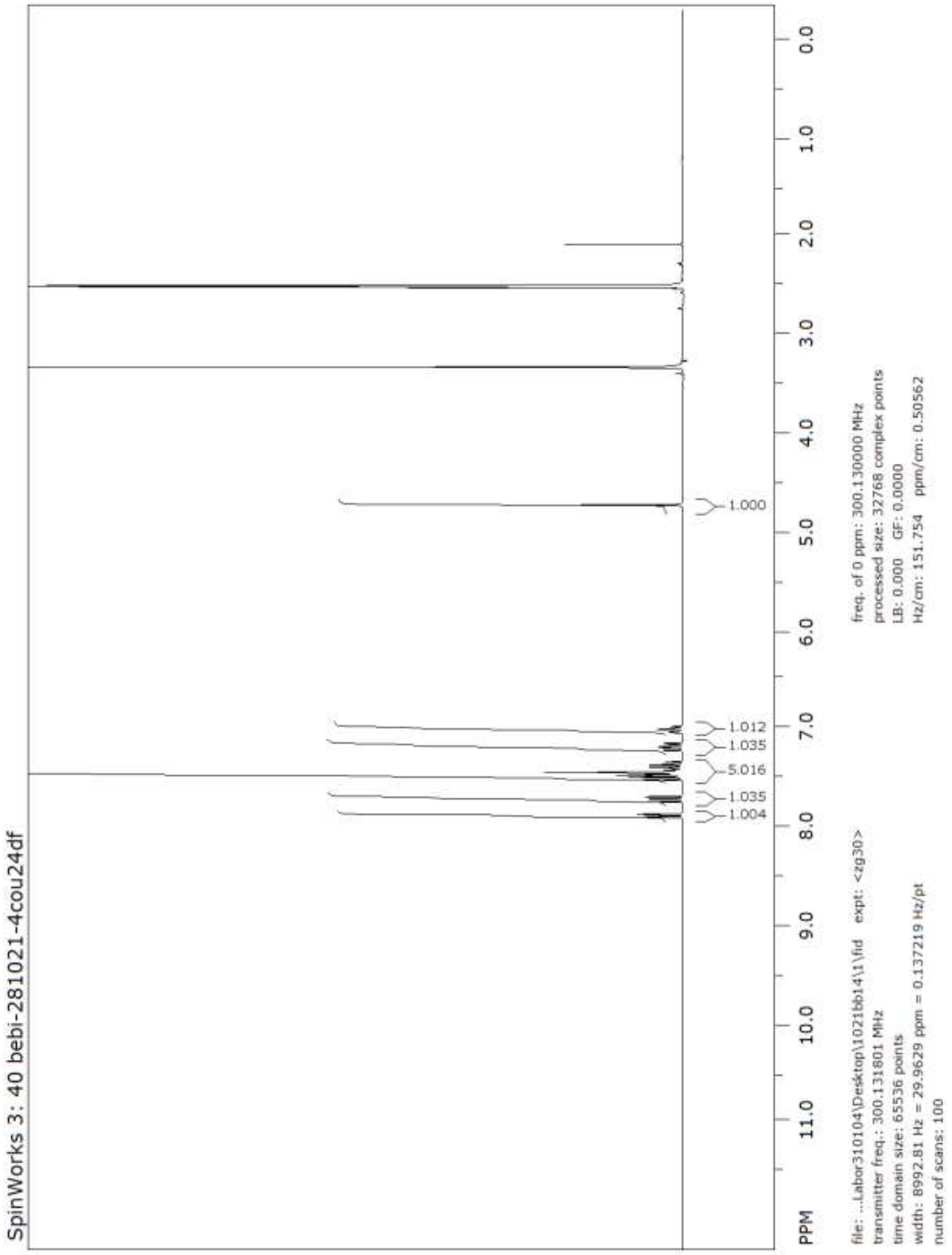

^{13}C NMR spectrum of **1p**

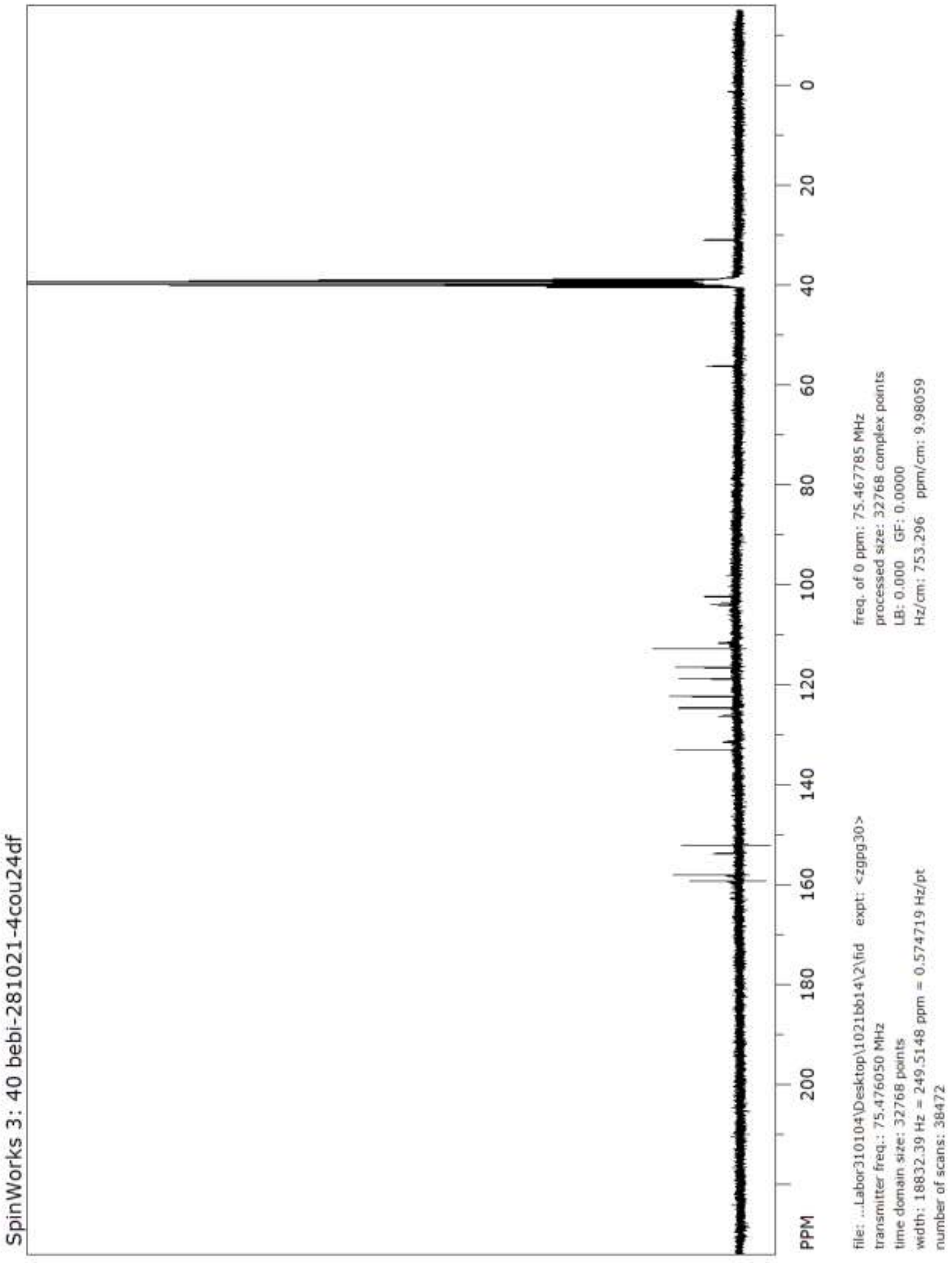

Results

Selectivity Index

To estimate their selectivity for cancer cells the three most active compounds **1a**, **1c**, **1d** were also tested on non-malignant adult human dermal fibroblasts (HDFa) via MTT-assays. The selectivity index (SI) for all tested cancer cell lines was calculated from the ratio of the average IC_{50} value and that of HDFa cells (Tab S1).

Table S1. Average IC_{50} value [µM] of all tested cancer cell lines the for compounds 1a, 1c and 1d and the calculated selectivity index (SI = IC_{50} nonmalignant HDFa cells / IC_{50} average of cancer cell lines). [1]

	Average IC_{50} value [µM]	Selectivity index (SI)
**1a**	3.41±1.5	29.3
**1c**	1.89±0.9	52.9
**1d**	3.21±1.0	31.1

Effects on the cell cycle

Effect of compounds **1a**, **1c**, **1d** as well as C-A4 and solvent (DMSO) on the cell cycle of 518A2 melanoma cells were assessed via PI staining and subsequent flow cytometry (Fig. S2).

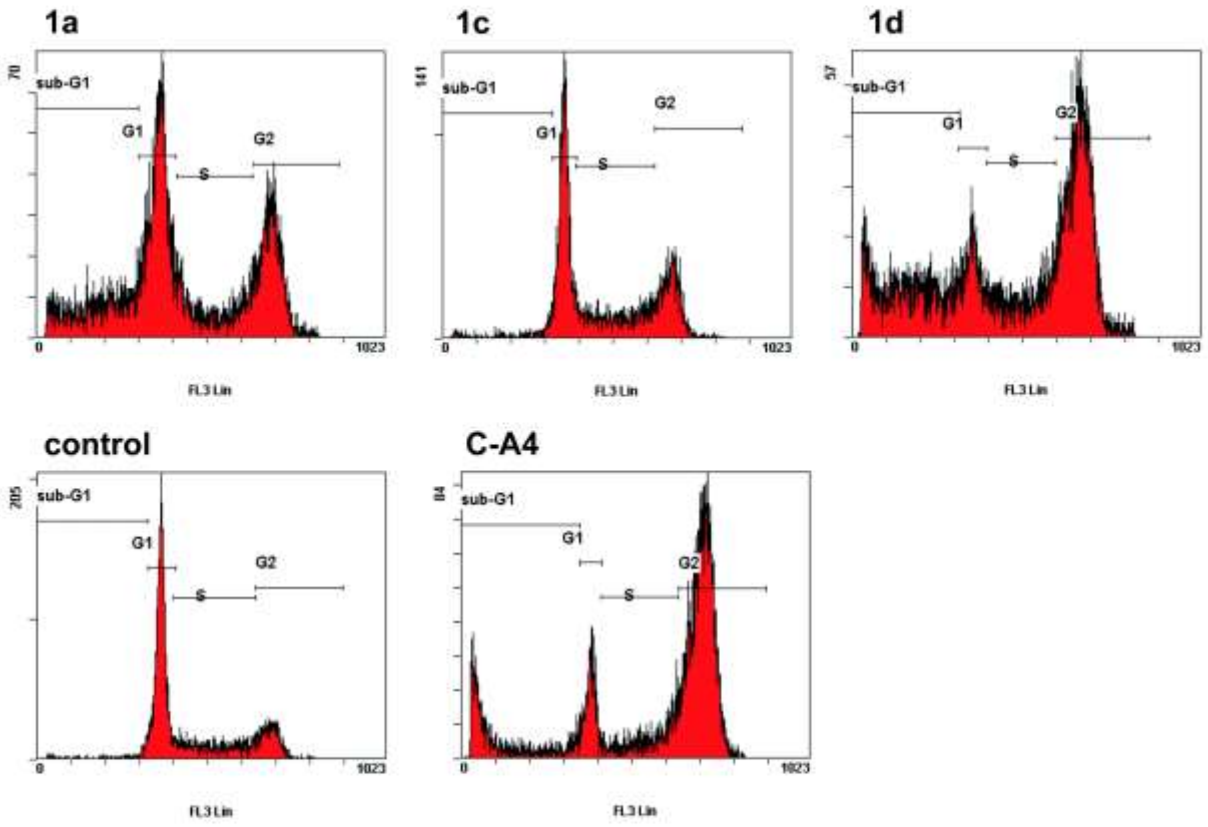

Figure S2. Representative cell cycle Histograms of 518A2 melanoma cells treated with 2.5 µM of **1a**, **1c**, **1d** or Combretastatin A4 (100 nM) for 12 h. Negative controls were treated with an equivalent amount of solvent (DMSO).

Caspase-3/7 activity

The activation of effector caspases 3 and 7 was investigated through an Apo-One® Homogenous Caspase-3/7 Assay Kit (Promega) after 6h of incubation with substances **1a**, **1c**, **1d** as well as C-A4 and vehicle DMSO (Fig S3).

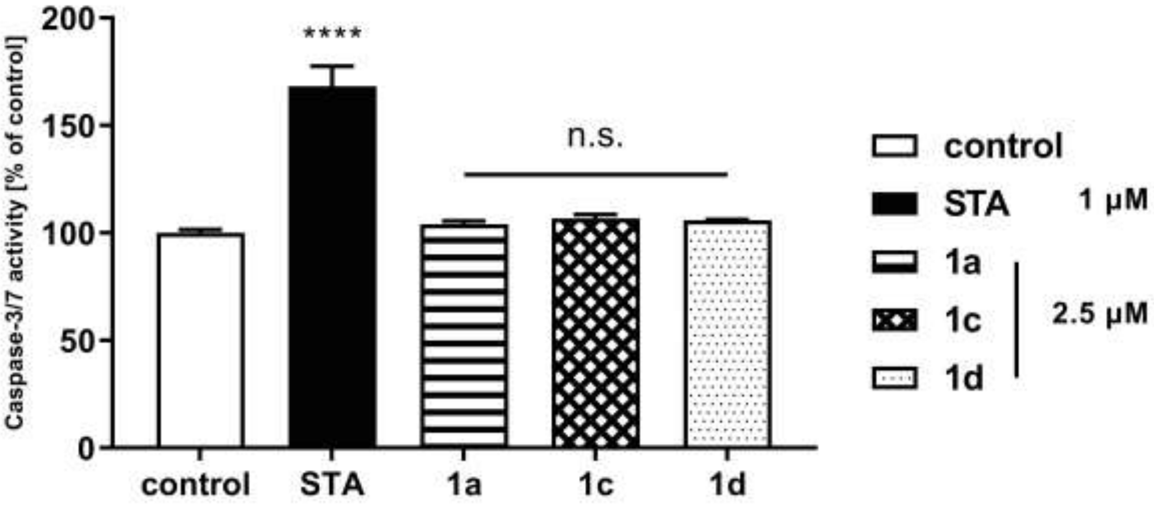

Figure S3. Measurement of caspase-3/7 activity via Apo-ONE® Homogenous Caspase-3/7 Assay Kit (Promega) after treatment of 518A2 melanoma cells with test compounds **1a**, **1c**, **1d** (2.5 µM) and staurosporine (1 µM). Solvent (DMSO) treated cells served as positive control and were set to 100%. Experiments were performed in triplicate and quoted as means ± SD. The significance was given as: ****: *p* <0.0001 against control, One-way ANOVA, with Dunnett's multiple comparison test (GraphPad Prism 7).

Tube-Formation viability (MTT-assay)

The vitality of the cells after treatment with **1a**, **1c**, **1d** as well as C-A4 and solvent (DMSO) for 3 h was determined by MTT assay to be higher than 80% compared to negative controls (Tab. S4).

Table S4. Viability of 518A2 melanoma cells after treatment with test compound 1a, 1c, 1d (1 and 2.5 µM) or C-A4 (100 nM) with vehicle (DMSO) treated cells set to 100%. Values were assessed by MTT-assay after 3 h of incubation under cell culture conditions.

	Concentration [µM]	Viability [% of control]
**control**	-	100.0
**C-A4**	0.1	81.1
**1a**	1.0	100.2
	2.5	106.9
**1c**	1.0	93.2
	2.5	81.0
**1d**	1.0	91.3
	2.5	84.7

References

[1] M. López-Lázaro, *Oncoscience* **2015**, *2*, 91.
